# STING Agonist‐Loaded Nanoparticles Promotes Positive Regulation of Type I Interferon‐Dependent Radioimmunotherapy in Rectal Cancer

**DOI:** 10.1002/advs.202307858

**Published:** 2023-12-08

**Authors:** Lei Wang, Han Zhou, Qingjing Chen, Zhiwen Lin, Chenwei Jiang, Xingte Chen, Mingdong Chen, Libin Liu, Lingdong Shao, Xiaolong Liu, Jianji Pan, Jingcheng Wu, Jibin Song, Junxin Wu, Da Zhang

**Affiliations:** ^1^ Department of Radiation Oncology Fujian Cancer Hospital Fujian Medical University Fuzhou 350025 P. R. China; ^2^ Department of Oncology the Second Affiliated Hospital of Nanchang University Nanchang 360000 P. R. China; ^3^ Department of Clinical Oncology The University of Hong Kong‐Shenzhen Hospital Shenzhen Guangdong 518053 P. R. China; ^4^ The United Innovation of Mengchao Hepatobiliary Technology Key Laboratory of Fujian Province Mengchao Hepatobiliary Hospital of Fujian Medical University Fuzhou 350025 P. R. China; ^5^ Department of Hepatopancreatobiliary Surgery First Affiliated Hospital of Fujian Medical University Fuzhou 350004 P.R. China; ^6^ School of Biomedical Engineering Shanghai Jiao Tong University Shanghai 200030 P. R. China; ^7^ Department of Radiation Oncology Mengchao Hepatobiliary Hospital of Fujian Medical University Fuzhou 350025 P. R. China; ^8^ CAS Key Laboratory of Design and Assembly of Functional Nanostructures Fujian Institute of Research on the Structure of Matter Chinese Academy of Sciences Fuzhou 350002 P. R. China; ^9^ Mengchao Med‐X Center Fuzhou University Fuzhou 350116 P. R. China; ^10^ Department of Health Science Technology and Education National Health Commission of the People's Republic of China Beijing 100088 China; ^11^ State Key Laboratory of Chemical Resource Engineering College of Chemistry Beijing University of Chemical Technology Beijing 10010 P. R. China

**Keywords:** cGAMP, hypoxia, platinum nanoparticles, radioimmunotherapy, radiosensitizers

## Abstract

Hypoxia‐associated radioresistance in rectal cancer (RC) has severely hampered the response to radioimmunotherapy (iRT), necessitating innovative strategies to enhance RC radiosensitivity and improve iRT efficacy. Here, a catalytic radiosensitizer, DMPtNPS, and a STING agonist, cGAMP, are integrated to overcome RC radioresistance and enhance iRT. DMPtNPS promotes efficient X‐ray energy transfer to generate reactive oxygen species, while alleviating hypoxia within tumors, thereby increasing radiosensitivity. Mechanistically, the transcriptomic and immunoassay analysis reveal that the combination of DMPtNPS and RT provokes bidirectional regulatory effects on the immune response, which may potentially reduce the antitumor efficacy. To mitigate this, cGAMP is loaded into DMPtNPS to reverse the negative impact of DMPtNPS and RT on the tumor immune microenvironment (TiME) through the type I interferon‐dependent pathway, which promotes cancer immunotherapy. In a bilateral tumor model, the combination treatment of RT, DMPtNPS@cGAMP, and αPD‐1 demonstrates a durable complete response at the primary site and enhanced abscopal effect at the distant site. This study highlights the critical role of incorporating catalytic radiosensitizers and STING agonists into the iRT approach for RC.

## Graphical abstract

DMPtNPS and radiotherapy (RT) have a potential to decrease tumor hypoxia and enhance radiosensitivity while provoking bidirectional regulatory effects on the immune response. Loading cGAMP on DMPtNPS reverse the negative impact on the immune response and improves immunotherapy through type 1 interferon pathway. When combined with DMPtNPS@cGAMP, RT and αPD‐1 therapy, the result is long‐lasting complete response and improved abscopal effects.

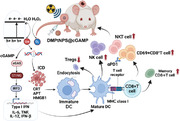

## 1 Introduction

Colorectal cancer (CRC) presents a considerable public health challenge in the United States. In 2020, there were an estimated 43340 new cases and 53200 deaths.^[^ ^1^ ^]^ Of all newly diagnosed CRC cases, rectal cancer (RC) accounts for 30%.^[^ ^2^ ^]^ In 2022, there are projected to be 44850 new cases of RC. Some improvements in morbidity have been achieved for RC management through cancer prevention and earlier diagnosis.^[^ ^3^ ^]^ However, the long‐term prognosis remains unfavorable, despite the promotion of complete mesorectal excision and the progression of systemic therapy, including immune checkpoint inhibitors (ICIs).

As a primary cancer treatment, radiotherapy (RT) is extensively utilized in various stages of RC, such as neoadjuvant RT for locally advanced RC, intraoperative RT, and salvage RT for advanced disease.^[^ ^3^ ^]^ Notably, RT has the ability to impact the tumor immune microenvironment (TiME), thereby demonstrating the potential for the combination of RT with immunotherapy (iRT).^[^ ^4^ ^]^ In a Phase II trial of pre‐RT followed by ICIs, the pathological complete response (pCR) reached as high as 48.1%,^[^ ^5^ ^]^ exceeding that of traditional neoadjuvant modalities for RC.^[^ ^6^ ^]^ Nevertheless, RT and iRT present several challenges. First, due to the moderate radiosensitivity of the RC, higher and potentially harmful radiation doses are often necessary. Secondly, hypofractionated RT can have a negative effect on the immune response by disrupting vascular permeability, inducing tissue fibrosis, exacerbating hypoxia, and activating myeloid‐derived suppressor cells and M2 macrophage infiltration.^[^ ^6^, ^7^ ^]^ The abscopal effect, which induces a systemic antitumor immune response outside the radiation site, remains rare even when combined with immunotherapy.^[^ ^6^, ^8^ ^]^ Thus, there is an urgent need for a novel RT approach to improve clinical outcomes.

High‐Z metals including gold,^[^ ^9^ ^]^ hafnium,^[^ ^10^ ^]^ selenium,^[^ ^11^ ^]^ and gadolinium‐^[^ ^12^ ^]^ based radiosensitizers, possess the ability to increase X‐ray permeability and retention. They can emit secondary electrons that can penetrate cells and hydrolyze water molecules, resulting in the generation of free radicals that cause DNA double‐strand breaks (DSBs) while minimizing side effects.^[^ ^10^ ^]^ These high‐Z radiosensitizers can enhance immunogenic cell death (ICD) mediated by RT and stimulate antitumor immunity.^[^ ^9^, ^10^, ^13^ ^]^ Compared to nanoparticles based on gold, hafnium, selenium, and gadolinium, the PtNPs based on Pt have unique features that enable more efficient transfer of X‐ray energy,^[^ ^13^ ^]^ resulting in the production of ROS and alleviation of hypoxia within tumors.^[^ ^14^ ^]^ However, hypofractionated RT‐induced immunosuppressive TiME cannot be reversed by PtNPs and the abscopal effect was unsatisfactory.^[^ ^15^ ^]^

Cyclic guanosine monophosphate‐adenosine monophosphate (cGAMP) has been identified as a second messenger that activates the cyclic GMP‐AMP synthase (cGAS)‐stimulator of interferon genes (STING) pathway within the TiME.^[^ ^16^ ^]^ This activation contributes to dendritic cell (DC) maturation, cytotoxic T lymphocyte (CTL) differentiation, and NK cell activation.^[^ ^16^ ^]^ RT activation of the cGAS‐STING pathway stimulates cGAMP generation, according to evidence.^[^ ^16^, ^17^ ^]^ However, the immunosuppressive TiME after RT^[^ ^4^, ^6^ ^]^ indicates that endogenous cGAMP is insufficient. Currently, administration of exogenous cGAMP could render the TiME susceptible to immunotherapy,^[^ ^17^, ^18^ ^]^ but the rapid clearance and degradation of unstable cGAMP limit its efficacy in vivo.^[^ ^19^ ^]^ To address these concerns, current nanotechnology developments have examined the transport of exogenous cGAMP through nanoparticles.^[^ ^10^, ^20^ ^]^ However, there exists limited research on cGAMP delivery for mitigating RT's adverse effects on TiME, necessitating immunotherapy.

**Scheme 1:**
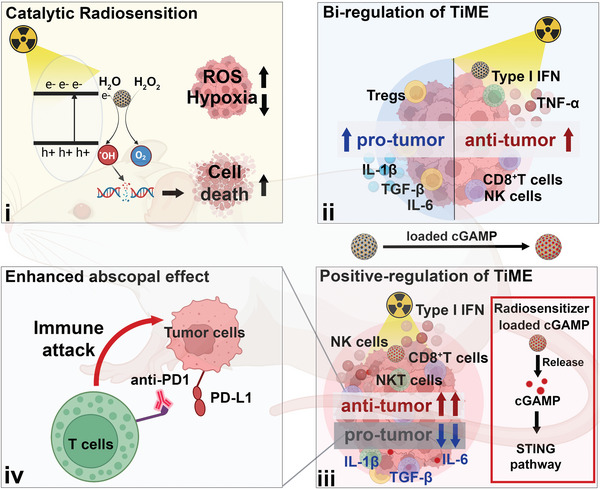
Preparation and mechanism of DMPtNPS and DMPtNPS@cGAMP. i. The catalytic radiosensitizer, DMPtNPS, sensitizes rectal cancer to radiotherapy (RT) by generating reactive oxygen species (ROS) and relieving hypoxia in tumors. ii. Catalytic radiosensitizer of DMPtNPS bi‐regulates the tumor immune microenvironment (TiME) during RT. iii. Delivery of exogenous cGAMP into the catalytic radiosensitizer (DMPtNPS@cGAMP) to make the TiME responsive to immunotherapy via activation of the stimulator of interferon genes (STING) pathway. iv. Combined with anti‐PD1 to enhance the abscopal effect of RT and DMPtNPS@cGAMP.

Here we synthesized DMPtNPS, a mesoporous silica catalytic radiosensitizer based on PtNPs, to enhance the radiosensitivity of RC by inducing the production of reactive oxygen species (ROS) and alleviating hypoxia in tumors. Nevertheless, upon a combination of DMPtNPS and RT, we observed a bidirectional TiME due to the activation of the cGAS‐STING pathway and induction of ICD. As a consequence, this diminished the beneficial RT‐induced immune response. We, therefore, incorporated exogenous cGAMP onto the DMPtNPS to further promote the STING‐mediated type I interferon signaling, resulting in enhanced antitumor immune responses and improved sensitivity to immunotherapy. In addition, we established an abscopal effect model to evaluate the efficacy of DMPtNPS@cGAMP in combination with iRT, providing a basis for a synergistic strategy to enhance iRT efficacy in future clinical translation (**Scheme** **1**).

## 2 Results

### 2.1 Preparation and Characteristics of DMPtNPS

**Figure 1:**
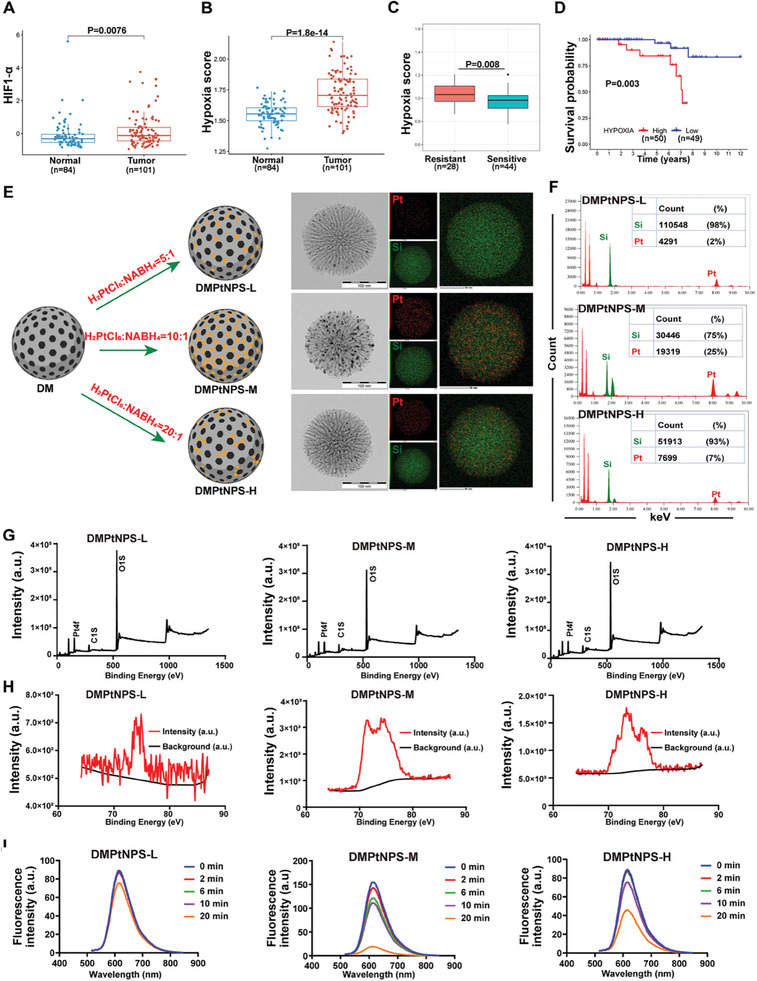
Preparation and characterization of DMPtNPS. A,B) Comparison of HIF‐1α mRNA expression and the single sample gene set enrichment analysis (ssGSEA) of hypoxia score between normal and rectal cancer tissues (normal, *n* = 84; tumor, *n* = 101). C) Comparison of hypoxia score ssGSEA between radioresistant and radiosensitive tissues (resistant, *n* = 28; sensitive, *n* = 44). D) Comparison of Kaplan‐Meier survival curves of patients with high and low hypoxia scores (hypoxia‐high, *n* = 50; hypoxia‐low, *n* = 49). E) Preparation of DMPtNPS‐L, DMPtNPS‐M, and DMPtNPS‐H were prepared using varying molar ratios of H_2_PtCl_6_ and NaBH_4_. F) The analysis of the compatible characteristic binding energy between silicon (Si) and platinum (Pt) in DMPtNPS‐L, ‐M, and ‐H. G) The Si and Pt element counts were quantified using energy dispersive X‐ray spectroscopy (EDS) in the three types of DMPtNPS. H) X‐ray photoelectron spectroscopy (XPS) was employed to analyze DMPtNPS‐L, ‐M, and ‐H. I) The fluorescence intensity of Ru(dpp)_3_Cl_2_ in DMPtNPS with hydrogen peroxide (H_2_O_2_, 100 × 10^−6^ m) at different co‐incubation times. ns indicates not significant, *P < 0.05, **P < 0.01. Data are presented as mean ± standard deviation (SD).

To examine the link between hypoxia and response to RT and prognosis, we evaluated data from the GSE56699, GSE233517, and GSE87211, including patients with RC who received RT. Our study found that the mRNA expression of hypoxia‐inducible factor‐1α (HIF‐1α) was significantly higher in CRC tissues than in normal tissues (*P* < 0.05, **Figure** **1**A). Through the single‐sample gene set enrichment analysis (ssGSEA), we identified 200 hypoxia‐related genes (Table S1, Supporting Information) and assessed each patient in both tumors and normal tissues. The hypoxia score was significantly higher in tumors compared to normal tissues (*P* < 0.05, Figure 1B). Additionally, RT‐sensitive tumors displayed a notably lower hypoxia score than that observed in RT‐resistant tumors (*P* < 0.05, Figure 1C). Furthermore, patients who presented high hypoxia scores exhibited a worse prognosis compared to those with a low score (*P* < 0.05, Figure 1D). These findings suggest that reducing hypoxia may be a promising strategy to reverse radioresistance and improve prognosis in RC.

To address this hypoxia, we developed a high‐Z catalytic radiosensitizer called DMPtNPS. This compound effectively catalyzes the production of oxygen (O_2_) from hydroperoxide and enhances the transfer of X‐ray energy to generate ROS. Initially, we synthesized dendritic mesoporous silica (DM) based on the previously reported.^[^ ^22^ ^]^ We then prepared DMPtNPS by in situ synthesis with H_2_PtCl_6_ and NaBH_4_. Different ratios of H_2_PtCl_6_ and NaBH_4_ were used to prepare DMPtNPS and optimize the dose of PtNPs. This resulted in the synthesis of three nanoparticles: DMPtNPS‐L, ‐M, and ‐H. TEM analysis indicated that DMPtNPS‐L, ‐M, and ‐H have average nanosizes of 93.4 ± 1.2, 96.9 ± 1.0, and 99.2 ± 1.6 nm, respectively. The zeta potentials of these nanoparticles were 30.2 ± 1.6, 34.5 ± 1.6, and 30.6 ± 1.4 mV, respectively (Figure 1E and Figure S1, Supporting Information). All samples exhibited similar Si and Pt bond energies, but DMPtNPS‐M displayed the highest Pt content ratio (25%) when compared to DMPtNPS‐L (2%) and DMPtNPS‐H (7%) (Figure 1F). The X‐ray photoelectron spectroscopy (XPS) analyses revealed a noticeably heightened PtNPs peak in DMPtNPS‐M (25318.93 eV) compared to the DMPtNPS‐L (5584.31 eV) and DMPtNPS‐H (15287.92 eV) (Figure 1G,H). In addition, we examined the O_2_ generation by DMPtNPS as it plays a crucial role in enhancing RT treatment.^[^ ^22^ ^]^ Following coincubation with H_2_O_2_ for 20 min, the fluorescence intensity of the O_2_ detector Ru(dpp)_3_Cl_2_ was significantly lower for DMPtNPS‐M than for DMPtNPS‐L and ‐H, suggesting that DMPtNPS‐M effectively catalyzed H_2_O_2_ to produce oxygen (Figure 1I). Significant differences in particle size, zeta potential, and O_2_ generation were observed between DMPtNPS and DM / DM‐NH_2,_ confirming our expectations (Figures S2 and S3, Supporting Information). Therefore, we used the DMPtNPS‐M for further study.

### 2.2 Radiation Sensitization of DMPtNPS In Vitro

**Figure 2:**
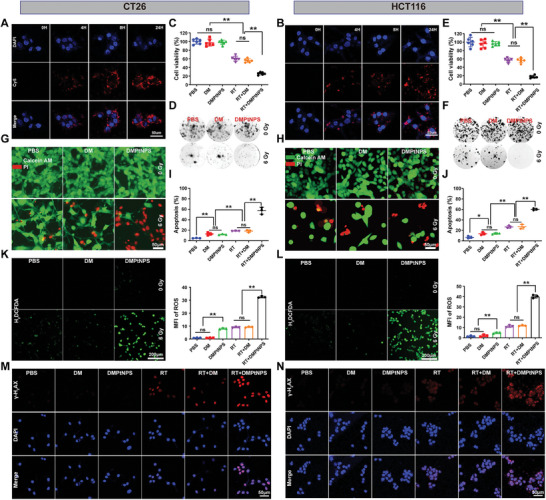
The in vitro radiosensitizing impact of DMPtNPS. A) CLSM images of CT26 cells and B) in HCT116 cells after coincubated with ^Cy5^DMPtNPS at different time points. C) Cell viability (*n* = 6) and D) clonogenic assay of CT26 cells after treatment with DM or DMPtNPS with or without X‐ray (6 Gy) irradiation for 48 h. E) Cell viability (*n* = 6) and F) clonogenic assay of HCT116 cells after treatment with DM or DMPtNPS with or without X‐ray (6 Gy) irradiation for 48 h. G) Calcein‐AM / PI staining of CT26 cells or H) HCT116 cells with or without X‐ray irradiation after co‐incubated with DM or DMPtNPS. Quantitative analysis of apoptotic cells in I) CT26 cells, *n* = 3 or J) in HCT116 cells, *n* = 3. CLSM images and quantitative analysis of intracellular ROS generation in K) CT26 cells, *n* = 3 and L) HCT116 cells, *n* = 3 after coincubated with PBS, DM, or DMPtNPS with or without X‐ray. Scale bar, 200 µm. M) CLSM images of N) γ‐H2AX in CT26 cells and in HCT116 cells after receiving different as indicated. ns indicates not significant, *P < 0.05, **P < 0.01. Data are presented as mean ± SD. Scale bar, 50 µm.

To evaluate the DMPtNPS uptake, we labeled DMPtNPS with Cy5‐NHS (^Cy5^DMPtNPS) and analyzed its uptake by confocal microscopy (CLSM) and flow cytometry (FCM) on murine CT26 and human HCT116 cell lines. Our results showed that ^Cy5^DMPtNPS localized to the cytoplasm of CT26 cells from 4 to 24 h in a time‐dependent manner (**Figure** **2**A,B). Similar results were observed in HCT116 cells. FCM data showed that at 4 h co‐incubation, approximately 89.0% ± 0.3% of CT26 and 84.6% ± 1.1% of HCT116 cells had effectively internalized ^Cy5^DMPtNPS (Figure S4, Supporting Information). The mean fluorescence intensity (MFI) of ^Cy5^DMPtNPS increased over time in both cell lines, and peaked at 24 h, further confirming the successful DMPtNPS internalization. Likewise, a time‐dependent uptake of DMPtNPS was also observed in human umbilical vein endothelial cells (HUVEC) (Figure S5, Supporting Information).

A Cell Counting Kit 8 (CCK8) assay was carried out to determine a safe but cytotoxic dose of DMPtNPS. The safe dose was determined to be 40 µg mL^−1^ for CT26 (94.6% ± 1.9% viable cells), HCT 116 (91.3% ± 2.8%), HUVEC (90.0% ± 1.0%), and DC2.4 (91.6 ± 5.7%) (Figure S6, Supporting Information). Notably, after exposure to 6 and 8 Gy doses of X‐ray irradiation, there was a sharp decrease in cell viability of CT26 and HCT116 when compared to the group treated with PBS. Specifically, the viability of CT26 cells was 59.9% and 64.1%, while that of HCT116 cells was 57.5% and 61.9%. However, no significant differences in cell viability were observed between 6 Gy and 8 Gy treatment (Figure S7, Supporting Information). Therefore, we selected the 6 Gy for further investigation.

To assess the radiosensitization capabilities of DMPtNPS, a series of experimentation were conducted which included CCK8, colony formation, live/dead cell staining, and apoptotic necrosis detection assays. Treatment with a combination of DMPtNPS (40 µg mL^−1^) and RT considerably decreased the number of viable CT26 and HCT116 cells to 25.7 ± 1.2% and 17.4 ± 1.3%, respectively, at 48 h compared to treatment with RT alone (Figure 2C,E). Similarly, the number of clones decreased in both cell lines when treated with a combination of therapies compared to RT alone (Figure 2D,F and Figure S8A,B, Supporting Information). Additionally, the ratio of dead‐to‐live cells significantly increased by using a combination treatment in both CT26 and HCT116 cells compared to RT alone (Figure 2G,H). Moreover, the combination treatment resulted in a notable increase in apoptosis with rates reaching 57.4 ± 3.8% in CT26 and 60.3 ± 1.2% in HCT116 cells compared to the RT alone (Figure 2I,J). These findings showed that DMPtNPS enhances the sensitivity of cell to RT and effectively induces radiation‐induced cytotoxicity.

Radiation causes tumor cell death by generating ROS that harm DNA.^[^ ^4^, ^11^ ^]^ To inspect the intracellular ROS formation, we applied the fluorescent probe 2′, 7′‐dichlorodihydrofluorescein diacetate (DCFH‐DA). After RT treatment, we discovered a high green fluorescence in CT26 and HCT116 cells treated with DMPtNPS compared to those with DM or RT treatment, with significantly increased MFI (Figure 2K,L). When DNA DSBs occur, phosphorylated H_2_AX (γ‐H_2_AX) rapidly forms large aggregates in cells.^[^ ^24^ ^]^ Therefore, we assessed levels of DSB and γ‐H2AX levels in cancer cells using immunofluorescence staining through CLSM and FCM analysis, respectively (Figure 2M,N and Figure S9A,B, Supporting Information). Our findings demonstrated that the DMPtNPS + RT group had higher γ‐H_2_AX levels than the RT alone group in both cell lines. In addition, γ‐H_2_AX levels were 2.3‐fold and 2.0‐fold higher in the DMPtNPS + RT group compared to the RT + DM group in both CT26 and HCT116 cells, respectively. These findings indicate that DMPtNPS enhances radiosensitization primarily by increasing ROS production.

### 2.3 Immune Cell Death Induced by DMPtNPS under RT In Vitro

**Figure 3:**
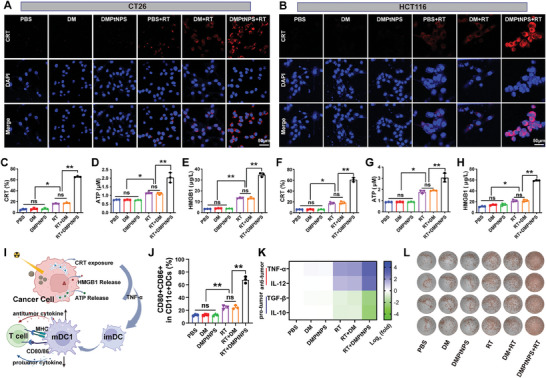
DMPtNPS enhanced RT‐induced immunogenic cell death. A) CLSM images of the exposure of calreticulin (CRT) in CT26 cells and B) in HCT116 cells after receiving different treatments as indicated, scale bar, 50 µm. C) Quantitative analysis of CRT exposure (*n* = 3), D) extracellular ATP levels (*n* = 3) and E) HMGB1 levels in CT26 cells after receiving different as indicated (*n* = 3). F) Quantitative analysis of CRT exposure (*n* = 3). G) extracellular ATP (*n* = 3), and H) HMGB1 levels in HCT116 cells after receiving different as indicated, *n* = 3. ns indicates not significant, *P < 0.05, **P < 0.01. Data are presented as mean ± standard deviation (SD). I) Schematic representation of DC maturation induced by ICD, resulting in CD8^+^T cell activation. J) Quantitative analysis of mature DCs (CD80^+^CD86^+^) in DC1 (CD11C^+^CD8a^+^) in the different treatment groups (*n* = 3). K) Quantitative analysis of cytokines levels associated with mature DCs in the different treatment groups. L) ELISpot analysis of IFN‐γ after receiving different treatments as indicated, *n* = 4.

ICD plays an important role in cancer immunotherapy.^[^ ^6b^ ^]^ To investigate the induction of ICD by DMPtNPS and RT treatment, the ICD biomarkers‐calreticulin (CRT), adenosine triphosphate (ATP), and high mobility group box 1 (HMGB1) were analyzed by immunofluorescence and FCM analysis, respectively. We found that significantly increased CRT exposure was observed in CT26 and HCT116 cells after DMPtNPS + RT treatment (**Figure** **3**A,B). Furthermore, FCM data showed that DMPtNPS + RT treatment significantly increased CRT^+^ cells to 65.8% ± 0.6% in CT26 and 60.9% ± 2.2% in HCT116 cells compared to RT alone (Figure 3C,F). Besides, extracellular ATP recruits and activates antigen‐presenting cells during ICD.^[^ ^24^ ^]^ We found that DMPtNPS + RT treatment significantly decreased the intracellular ATP and increased ATP release to the extracellular matrix compared to DM + RT or RT treatment alone in both cell lines (Figure 3D,G and Figure S10, Supporting Information). Further, HMGB1 promotes DC maturation, T‐cell activation, and pro‐inflammatory cytokines during ICD.^[^ ^24^, ^25^ ^]^ We also observed that the DMPtNPS + RT treatment increased HMGB1 levels (2.6‐fold in CT26 and 2.7‐fold in HCT116 cells compared to RT treatment alone (Figure 3E,H). These results suggest that DMPtNPS efficiently enhances RT‐induced ICD in CT26 and HCT116 cells.

Enhanced ICD then promotes the maturation of DCs.^[^ ^26^ ^]^ To test this, we treated CT26 cells with DMPtNPS + RT and then co‐cultured them with bone marrow‐derived DCs (BMDCs) for 24 h (Figure 3I). FCM data showed that the DMPtNPS + RT treatment significantly increased the proportion of mature DCs (mDCs) within the DC1 population to 67.8% compared to 24.2% with RT alone and 24.6% with DM + RT group (Figure 3J). In addition, the secretion of interleukin‐12 (IL‐12) and tumor necrosis factor‐α (TNF‐α) from mDCs promotes the differentiation of helper T cell (Th0) cells into Th1 cells, thereby triggering cellular immunity.^[^ ^27^ ^]^ Conversely, interleukin‐10 (IL‐10) and transforming growth factor beta (TGF‐β) can induce the regulatory T cells (Tregs) differentiation, leading to immune tolerance.^[^ ^27^ ^]^ As expected, the DMPtNPS + RT group increased TNF‐α and IL‐12 by 2.4‐fold and 26‐fold over RT alone but decreased TGF‐β and IL‐10 by about 3.1‐fold and 3.7‐fold, respectively (Figure 3K and Figure S11, Supporting Information). These findings imply that the combination of DMPtNPS and RT has the ability to regulate the DC1 response by boosting pro‐inflammatory cytokines and reducing anti‐inflammatory cytokines.

Upon activation, CTLs can eliminate tumor cells by producing antitumor cytokines like IFN‐γ.^[^ ^26b^ ^]^ We evaluated IFN‐γ levels in activated CD8^+^T cells using an ELISPOT kit and observed that the DMPtNPS + RT group had 2.2‐fold increased IFN‐γ spots per 10^5^ cells compared to RT alone (Figure 3L and Figure S12, Supporting Information). These findings suggest that DMPtNPS enhances the RT‐induced ICD and effectively initiates a DC‐CTL antitumor immune response.

### 2.4 Biosafety and Radiosensitization of DMPtNPS In Vivo

**Figure 4:**
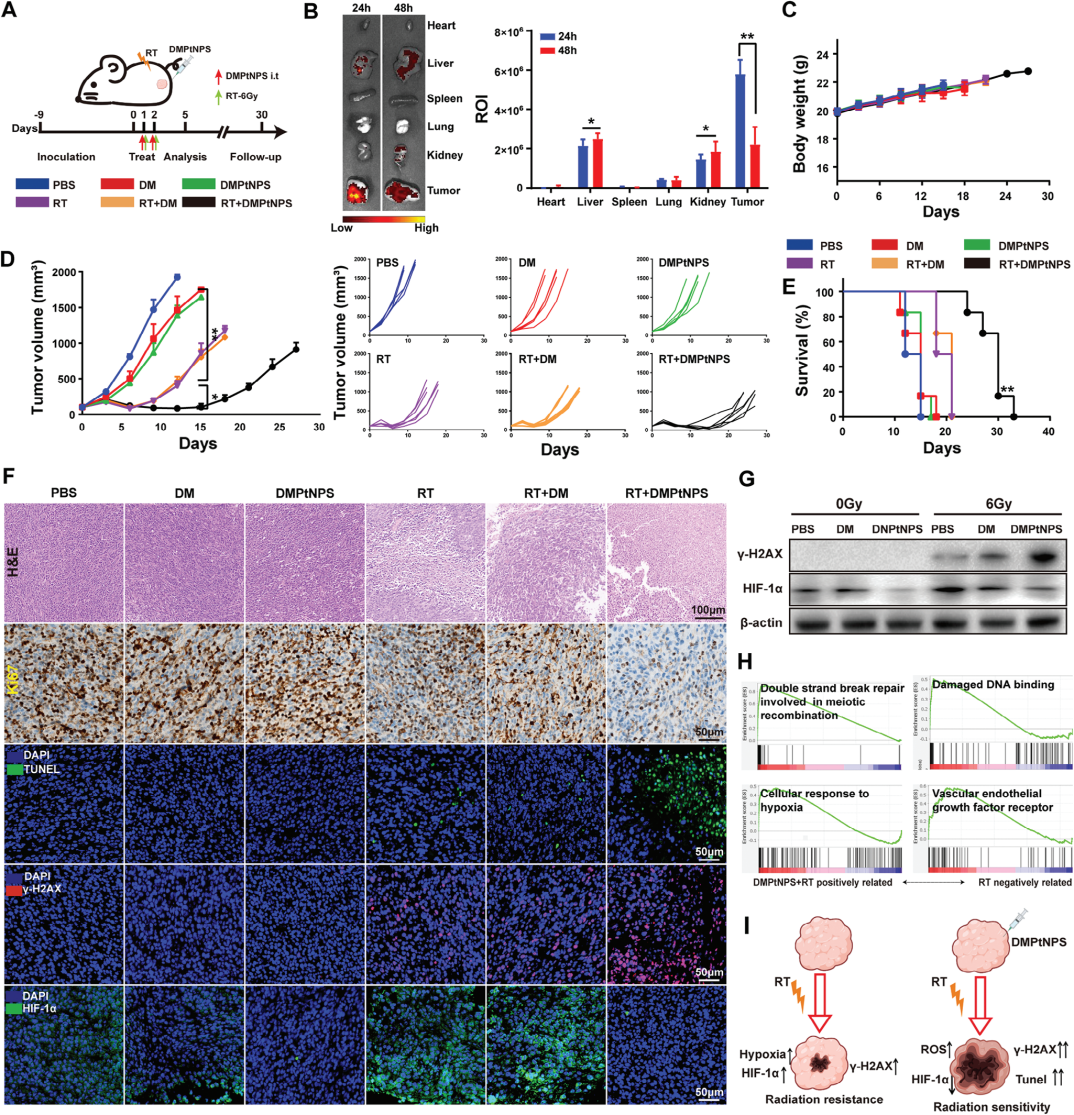
In vivo radiosensitizing effect of DMPtNPS. A) Evaluation of in vivo radiosensitization effect of DMPtNPS using a CT26‐bearing mouse model. B) Ex vivo fluorescence imaging and quantitative analysis of major organs dissected from mice after intravenous injection of DMPtNPS at 24 and 48 h (*n* = 3). *C*) Measurement of body weight loss in CT26‐bearing mice after receiving different treatments (*n* = 6 mice per group). D) Tumor volume curve of mice after receiving PBS, DM, or DMPtNPS with or without X‐ray (6 Gy) (*n* = 6 mice per group). E) Kaplan–Meier survival curves of mice after receiving different treatments (*n* = 6 mice per group). F) Histological examination of tumor tissues using H&E staining (scale bar, 100 µm), Ki67 staining, immunofluorescence analysis of TUNEL (green), γ‐H2AX (red), and HIF‐1α (green) at day 5 after receiving different treatments. Scale bar, 50 µm. G) Western blot analysis of γ‐H2AX and HIF‐1α at day 5 after receiving different treatments. H) GSEA is based on differentially expressed genes between the DMPtNPS + RT group and the RT group (*n* = 4 mice per group). I) Schematic diagram illustrating the radiosensitizing effect of DMPtNPS. ns indicates not significant, *P < 0.05, **P < 0.01. Data are presented as mean ± SD.

Encouraged by the in vitro findings, we assessed the radiotherapeutic efficacy of DMPtNPS in vivo (**Figure** **4**A). To minimize the damage to healthy tissues attributable to RT therapy, we administered DMPtNPS intratumorally rather than intravenously.^[^ ^20^, ^28^ ^]^ Using a UniNano NIR‐II In Vivo Imaging System, we imaged the organs and tumors of CT26 tumor‐bearing mice after intratumoral injection of ICG‐NHS labeled DMPtNPS (^ICG^DMPtNPS). At 24 h postinjection, ^ICG^DMPtNPS exhibited greater fluorescence intensity in the tumors than in the liver (Figure 4B). In addition, there were no obvious signals detected in the heart, spleen, or kidneys, indicating there was a swift accumulation and clearance of the tumor. However, increases in liver and kidney signals at later time points suggested that there were hepatic and renal clearance pathways. These results indicate that DMPtNPS accumulates effectively in tumors for at least two days after injection, before being cleared eliminated by the liver and kidneys.

As expected, this study revealed that DMPtNPS effectively delayed the regrowth of tumors induced by RT when compared to other treatment groups. Specifically, on day 12, the DMPtNPS+ RT group demonstrated a significant reduction in tumor volume as compared to those undergoing RT alone or receiving DM + RT treatments (*P <* 0.05) (Figure 4D). Moreover, the DMPtNPS + RT group exhibited a median overall survival (OS) of 30 d, a significant improvement when compared with RT alone (19.5 d) or DM + RT groups (21 d) (Figure 4E). Furthermore, the augmented tumor necrosis led to a decrease in Ki67 staining and an expansion in TUNEL staining areas, thereby confirming the synergistic effect of DMPtNPS + RT treatment (Figure 4F). These outcomes establish that DMPtNPS augments tumor response and enhances survival rates in mice after radiotherapy.

Next, we investigated γ‐H_2_AX levels in tumors using immunofluorescence and western blot. We observed that the staining of γ‐H_2_AX was higher in the DMPtNPS + RT group as compared to the RT + DM group or only RT treated group (Figure 4F). Furthermore, the analysis revealed that γ‐H2AX expression was upregulated in the DMPtNPS + RT group, indicating efficient DSB enhancement by DMPtNPS and RT when compared to DM + RT or RT treatment alone (Figure 4G). Tumor hypoxia can cause radioresistance, making improved oxygenation a potential pathway to enhance radiosensitization.^[^ ^26^, ^29^ ^]^ Immunofluorescence and western blot analyses show reduced tumor HIF‐1α levels with DMPtNPS compared to DM‐treated groups, with or without RT (Figure 4F,G). This downregulation was attributed to the catalase activity of DMPtNPS. These results support DMPtNPS's efficacy in reducing tumor hypoxia and enhancing radiosensitization of RC.

To gain insight into the potential mechanism of DMPtNPS as a radiosensitizer, transcriptome sequencing was performed on the RT+ DMPtNPS versus RT alone. We identified 101 differentially expressed genes (DEGs) with DMPtNPS + RT group compared to RT alone (Table S2, Supporting Information). Among these genes, 88 genes were upregulated while 13 were downregulated (Figure S13A, Supporting Information), and further illustrated in a heat map (Figure S13B, Supporting Information). GSEA analysis showed that pathways related to DNA DSB, damaged DNA binding, cellular hypoxia response, and vascular endothelial growth factor receptor regulation were significantly enriched in the DMPtNPS + RT group compared to RT alone (Figure 4H). These results provided further evidence that DMPtNPS improves tumor radiosensitization by alleviating hypoxia in vivo (Figure 4I).

In addition, biosafety evaluations of DMPtNPS were conducted through monitoring of body weight and biochemical indices post‐treatment. There were no significant differences in the body weight of the mice among the various treatment groups (Figure 4C). Moreover, assessments of liver enzymes (ALT, AST), and kidney function (CREA, UREA) displayed no noteworthy differences among the different treatment groups (Figure S14A–D, Supporting Information). The histological analysis indicated no changes in the heart, liver, spleen, lung, and kidney among the various treatment groups, providing partial confirmation of the biosafety of DMPtNPS (Figure S14E, Supporting Information).

### 2.5 Biregulation of TiME by DMPtNPS In Vivo

Nondurable tumor response, characterized by initial shrinkage followed by eventual progression due to ineffective therapy, is a concern with immune/radiotherapy.^[^ ^30^ ^]^ Our study found that all mice receiving RT and DMPtNPS did not achieve CR and instead displayed retaliatory tumor growth after a transient treatment response. Simply increasing radiation sensitivity is insufficient to achieve a durable CR. The non‐durable response to RT and DNA‐damaging DMPtNPS is likely due to the complex, dynamic TiME.

**Figure 5:**
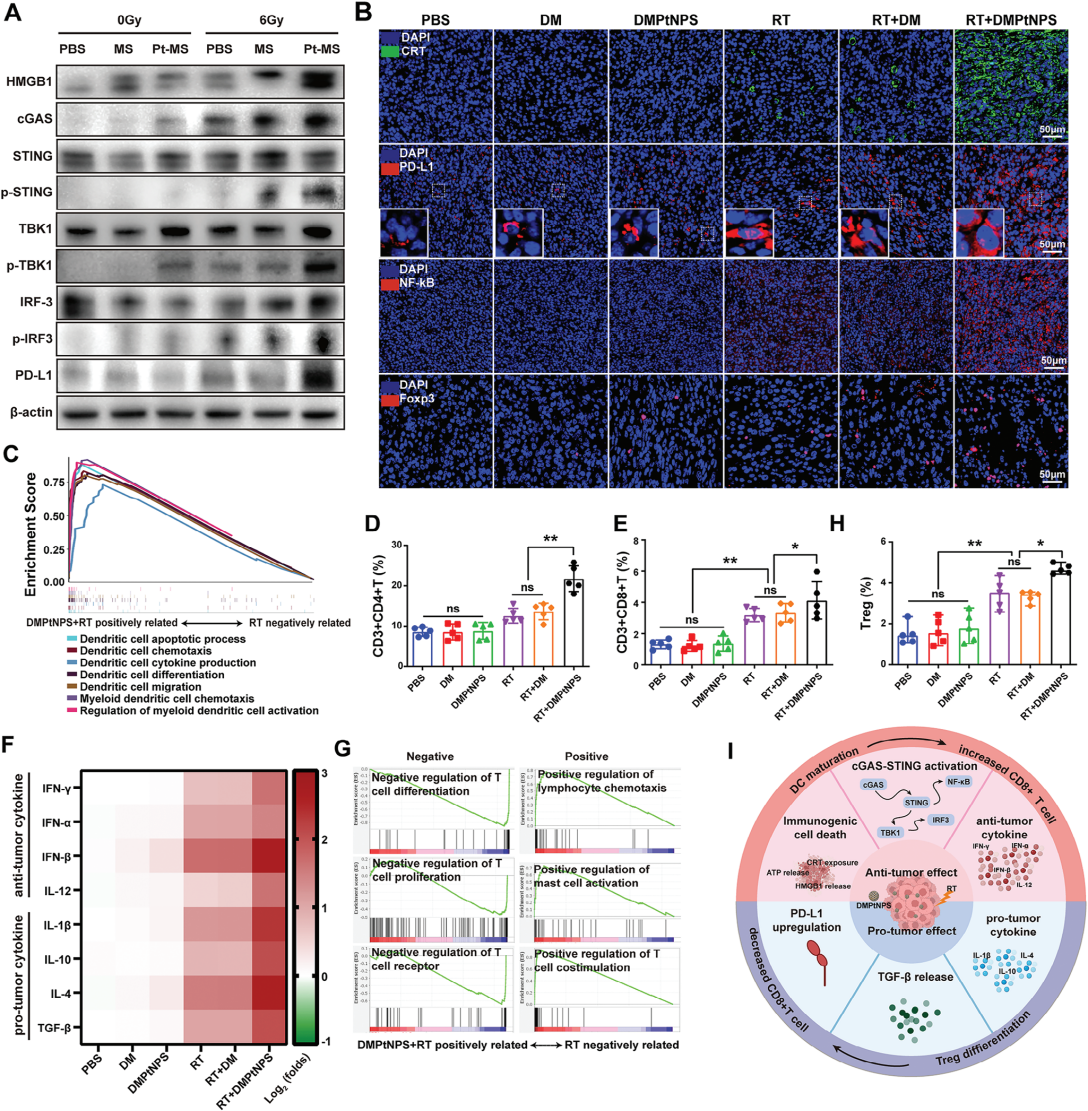
Biregulation of TiME induced by DMPtNPS and RT. A) Western blot analysis of HMGB1, cGAS, STING, phosphorylated‐STING, TBK1, phosphorylated‐TBK1, IRF3, phosphorylated‐IRF3, and PD‐L1 at day 5 after receiving different treatments. B) Immunofluorescence analysis of CRT (green), PD‐L1 (red), NF‐κB (red), and Foxp3 (red) on day 5 after receiving different treatments. Scale bar, 50 µm. C) Enrichment of DC‐related pathway in DMPtNPS + RT group compared to the RT alone as determined by GSEA analysis. Flow cytometry analysis of D) tumor‐infiltrating CD4^+^T cells (*n* = 5), E) CD8^+^T cells (*n* = 5). F) Heatmap showing the levels of anti‐tumor and pro‐tumor cytokines in tumor tissues from different treatment groups (*n* = 5). G) Enrichment of positive and negative immune response based on GSEA analysis in DMPtNPS + RT and RT alone (*n* = 4). H) Flow cytometry analysis of Tregs (CD25^+^Foxp3^+^) in tumors (*n* = 5). ns indicates not significant, *P < 0.05, **P < 0.01. Data are presented as mean ± SD. I) Schematic illustration of the biregulation of TiME induced by DMPtNPS and RT.

Compared to the findings from in vitro experiments, western blots analysis showed an enhanced ICD effect with upregulated tumor HMGB1 expression in DMPtNPS + RT group compared to other treatment groups (**Figure** **5**A and Figure S15A, Supporting Information). Furthermore, immunofluorescence demonstrated an increased CRT staining in the DMPtNPS + RT group compared to the other groups, thus confirming an increased ICD (Figure 5B). As previously reported,^[^ ^31^ ^]^ a combination of RT with DMPtNPS resulted in significant activation of the cGAS‐STING pathway. This was evidenced by the upregulation of cGAS, p‐STING, phosphorylated‐TANK binding kinase 1 (p‐TBK1), phosphorylated‐interferon regulatory factor 3 (p‐IRF3), and increased nuclear factor kappa‐B (NF‐κB) staining. Notably, downstream of the cGAS‐STING pathway,^[^ ^16^, ^31^ ^]^ programmed death ligand 1 (PD‐L1) was also upregulated following treatment with DMPtNPS and RT, as detected by western blot and immunofluorescence (Figure 5A,B and Figure S15B, Supporting Information).

In addition, the generation of ICD and activation of the cGAS‐STING pathway have the potential to enhance the maturation of DCs and stimulate anti‐tumor immunity in vivo.^[^ ^16^, ^24^ ^]^ In this study, the bioinformatics analysis disclosed a higher enrichment of DC‐related pathways such as cytokine signaling, chemotaxis, differentiation, and migration with DMPtNPS + RT compared to RT alone (Figure 5C). The FCM results demonstrate a population increase of DC1 and mDCs increased to 0.37% ± 0.22% and 21.94% ± 15.8%, respectively, in the DMPtNPS + RT treatment group compared to the RT group (0.22% ± 0.03% and 15.8% ± 1.1%) (Figure S16, Supporting Information). We also evaluated changes in the TiME by analyzing tumor‐infiltrating CD3^+^CD4^+^ and CD8^+^ T cells. The FCM data showed that the DMPtNPS + RT treatment resulted in a higher percentage of CD3^+^CD4^+^ and CD3^+^CD8^+^T cells compared to RT or DM + RT treatment alone (Figure 5D,E). Furthermore, we observed a significantly increased in antitumor cytokines including IFN‐γ, IFN‐α, IFN‐β, and IL‐12 by 3.1‐, 2.7‐, 6.1‐, and 3.1‐fold compared to PBS, respectively, in DMPtNPS + RT treatment group compared to PBS (Figure 5F and Figure S17A–D, Supporting Information). These findings suggest that DMPtNPs may enhance antitumor immunity.^[^ ^27^ ^]^ In addition, GSEA analysis showed that DMPtNPS + RT treatment positively regulated immune pathways such as lymphocyte chemotaxis, mast cell activation, and T cell co‐stimulation, compared to RT alone (Figure 5G). These results provide evidence that DMPtNPS is capable of efficiently enhancing the RT‐induced immune response in vivo.

Nevertheless, RT has both positive and negative effect, including the induction of immunosuppressive factors.^[^ ^6^, ^7^ ^]^ Our study showed that the use of DMPtNPS + RT resulted in a 1.4‐fold increase in Tregs, which are known to facilitate tumor progression and immune evasion,^[^ ^32^ ^]^ compared to RT alone (Figure 5H). Moreover, cytokines such as IL‐1β, IL‐10, IL‐4, and TGF‐β also increased (Figure 5F and Figure S17E–H, Supporting Information), which may promote tumor growth through inflammation.^[^ ^27^ ^]^ In addition, GSEA analysis demonstrated that the DMPtNPS + RT group had enrichment of pathways associated with negatively regulating T cell differentiation, proliferation, and TCR signaling compared to RT alone using GSEA. These results suggest that the combination treatment of RT and DMPtNPS has a detrimental impact on the TiME, further supporting the modification of catalytic radiosensitizer (Figure 5I).

### 2.6 DMPtNPS@cGAMP Enhanced Positive Regulation of TiME under the RT Treatment

**Figure 6:**
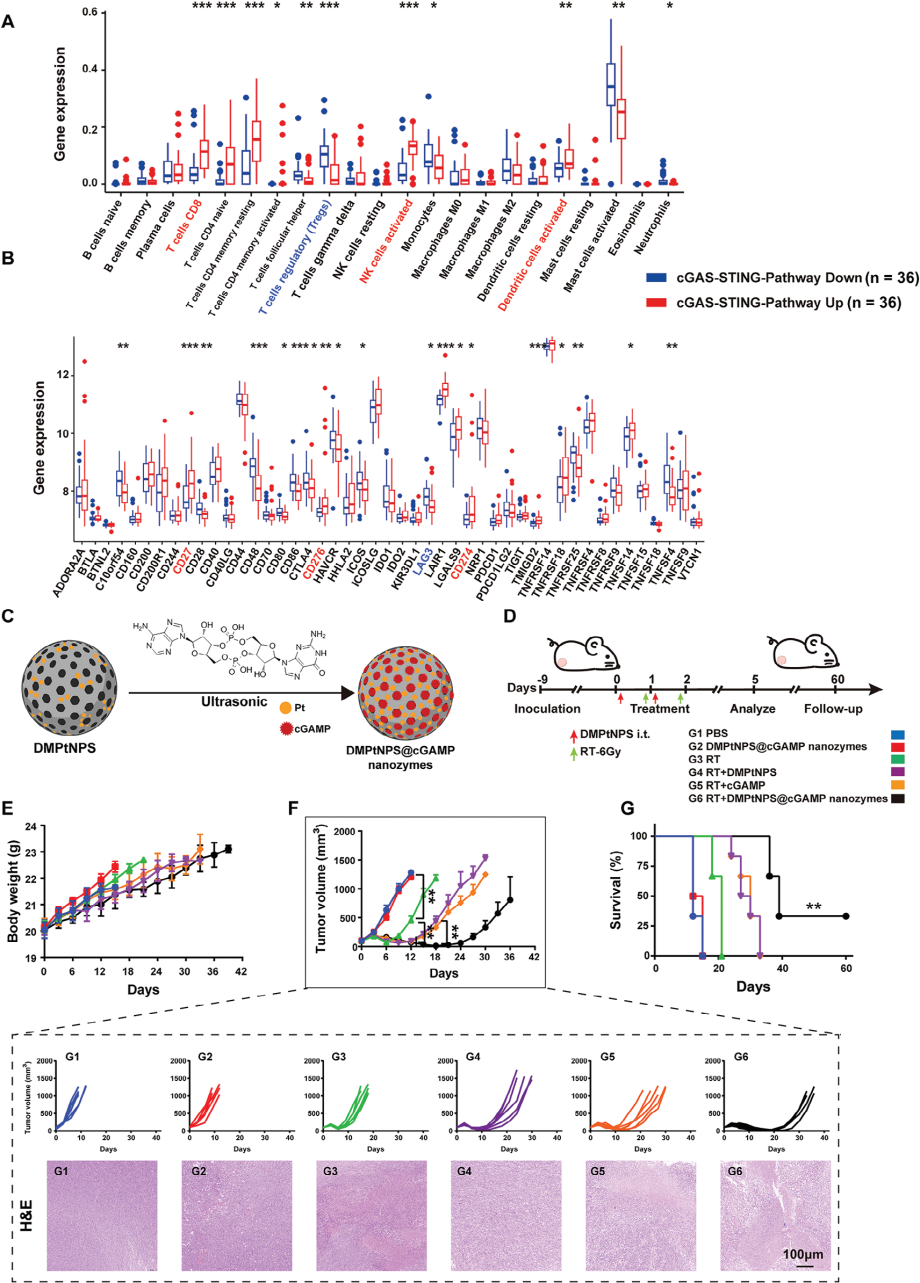
Integration of cGAMP into DMPtNPS to positively regulate the TiME under in vivo radiotherapy. A) Comparative analysis of immune cell populations for both down‐regulated and up‐regulated cGAS‐STING pathway via the “CIBERSORT” R package (down, *n* = 36; up, *n* = 36). B) Comparative analysis of immune checkpoint genes for both down‐regulated and up‐regulated cGAS‐STING pathways (down, *n* = 36; up, *n* = 36). C) Schematic diagram illustrating the synthesis of DMPtNPS@cGAMP. D) The process of in vivo antitumor effect of DMPtNPS@cGAMP in CT26‐bearing tumor mouse model. E) Body weight loss of mice after receiving different treatments as indicated (*n* = 6). F) Tumor volume curve of mice after receiving different treatments as indicated, (*n* = 6), and H&E staining on the day 5 after treatment, scale bar, 100 µm. G) Kaplan‐Meier survival curves of mice after different treatments as indicated, (*n* = 6). ns indicates not significant, *P < 0.05, **P < 0.01. Data are presented as mean ± SD.

The STING agonist cGAMP facilitates DC polarization, CD8^+^T cell differentiation, and NK cell activation, leading to a potent antitumor response.^[^ ^33^, ^34^ ^]^ To investigate the impact of the cGAS‐STING pathway on the TiME, we used GSE56699 to analyze the CIBERSORT data.^[^ ^35^ ^]^ On the one hand, Tregs showed the opposite pattern (**Figure** **6**A). The results indicated an increase in CD8^+^T cells, NK cells, and DCs with upregulation of cGAS‐STING pathway as compared to its downregulation. In addition, we compared immune checkpoint genes using the cGAS‐STING pathway score (Figure 6B). As expected, PD‐L1 expression exhibited a positive correlation with the cGAS‐STING pathway, together with the costimulatory molecules CD27 and CD276 that stimulate cellular immunity.^[^ ^36^ ^]^ Conversely, LAG3 abundance showed a negative correlation with the cGAS‐STING pathway, which was found to negatively regulate MHC class II‐restricted T‐cell responses^[^ ^37^ ^]^ but is coexpressed on exhausted CD8^+^T cells.^[^ ^38^ ^]^ Taken together, cGAMP could potentially serve as an effective approach for counteracting the inhibitory TiME triggered by RT and DMPtNPS.

We confirmed our hypothesis by using exogenous cGAMP onto DMPtNPS (Figure 6C). DMPtNPS's inherent pores allowed for efficient cGAMP loading. A TEM image confirmed the uniform nanosphere morphology of DMPtNPS@cGAMP (110.76 ± 10.90 nm), with a reduced zeta potential (11.10 ± 1.2 mV) compared to DMPtNPS (Figure S18A,B, Supporting Information). The EDS mapping also identified signals of loaded cGAMP (P), Si, O, and Pt from PtNPS, indicating successful encapsulation (Figure S18C, Supporting Information). Additionally, we found that the encapsulation efficiency, determined by using high‐performance liquid chromatography (HPLC), was 12.8 wt% cGAMP (Figure S18D, Supporting Information). Firstly, the acidic‐responsive release of cGAMP in DMPtNPS@cGAMP was investigated under various pHs (7.4, 6.5 and 5.0). The results showed that DMPtNPS@cGAMP achieved a drug release rate of up to 57.38% after 120 h of coincubation in PBS buffer under pH 7.4 (Figure S19, Supporting Information). In contrast, at pH 5.0 or 6.5, the drug release rates increased to 81.26% and 65.04% respectively, indicating the sustained‐release and acidic‐response ability of our prepared DMPtNPS@cGAMP. Next, we evaluated the in vitro antitumor effect of DMPtNPS@cGAMP. In comparison to radiation therapy (RT) alone (51.33% ± 5.90% and 46.80% ± 3.17%), DMPtNPS@cGAMP significantly increased toxicity by 65.8% in CT26 cells and 72.3% in HCT116 cells after RT treatment (Figure S20, Supporting Information). Furthermore, in CT26 cells, DMPtNPS@cGAMP significantly enhanced the levels of γ‐H2AX by 29.6% compared to RT alone (96.32 ± 1.01% vs 74.33 ± 1.46%) (Figure S21, Supporting Information). Additionally, when compared to treatment with RT alone, DMPtNPS@cGAMP significantly increased the exposure of CRT, as well as the release of HMGB1 and ATP, leading to approximately 2.5‐fold, 1.7‐fold, and 1.7‐fold increases, respectively. Comparable results were observed in HCT116 cells after treatment with DMPtNPS@ cGAMP and RT (Figure S22, Supporting Information). Therefore, we further investigated the antitumor effect of DMPtNPS@cGAMP and RT in a mouse model with CT26 tumors (Figure 6D). Throughout the treatment, no differences in body weight were observed among the groups (Figure 6E). On day 18, the tumor volumes were significantly lower in the RT + DMPtNPS@cGAMP group compared to the RT + DMPtNPS or RT + cGAMP group (Figure 6F). Notably, 2 out of 6 mice achieved a durable CR in the DMPtNPS@cGAMP + RT group. H&E staining revealed elevated tumor necrosis in the DMPtNPS@cGAMP + RT group compared to other groups. Further, survival analysis indicated the the benefit of DMPtNPS@cGAMP + RT group, with a prolonged median OS of 39 d compared to 28.5 d in the DMPtNPS + RT group (Figure 6G). These results suggest that the combination of DMPtNPS@cGAMP and RT not only curbed tumor growth but also resulted in long‐lasting tumor suppression.

**Figure 7:**
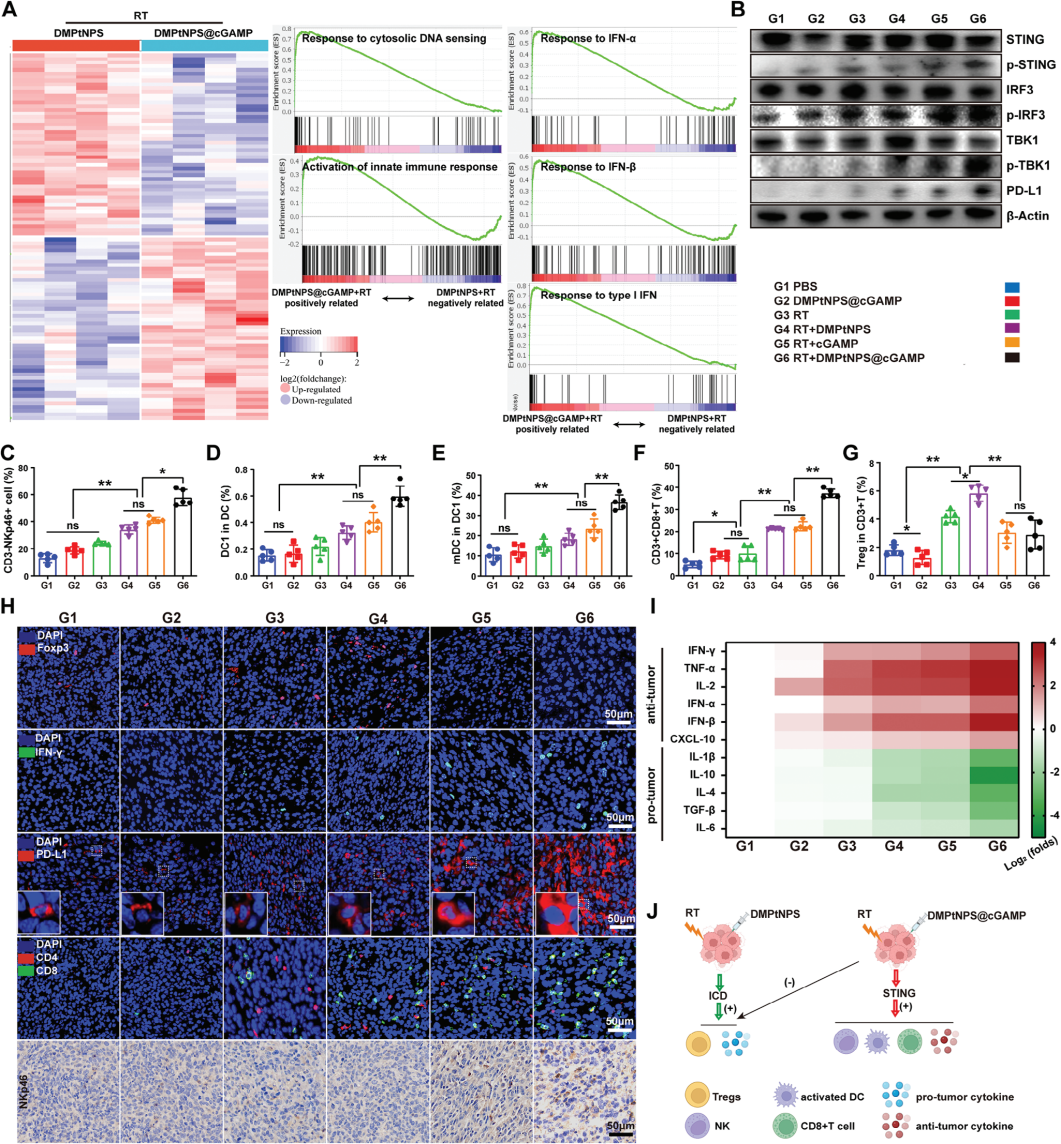
A combination of DMPtNPS@cGAMP and RT positively remodeling the TiME. A) DEGs between the DMPtNPS+RT group and the DMPtNPS@cGAMP+RT group. GSEA analysis showing the cGAS‐STING pathway‐related function based on the DEGs (*n* = 4). B) Western blot analysis of cGAS‐STING pathway‐related proteins on day 5 after different treatments as indicated. Flow cytometry of C) tumor‐infiltrating NK cells (CD3^−^NKp46^+^) (*n* = 5), D) DC1 (CD11C^+^CD8a^+^) (*n* = 5), E) mDCs (CD80^+^CD86^+^) (*n* = 5), F) CD8^+^T cells (*n* = 5) and G) Tregs (CD25^+^Foxp3^+^) (*n* = 5). H) Typical images of Foxp3 (red), IFN‐γ (green), PD‐L1 (red), CD4 (red), and CD8 (green) on day 5 after treatment. Scale bar, 50 µm. I) Heatmap in this figure represents levels of suppressive cytokines and active cytokines in tumor tissues from different treatment groups (*n* = 5). J) The schematic diagram showing the positive regulation in TiME caused by DMPtNPS@cGAMP and RT. ns indicates not significant, *P < 0.05, **P < 0.01. Data are presented as mean ± SD.

We evaluated the changes in the TiME following treatment with RT and DMPtNPS@cGAMP. Transcriptomics analysis identified 105 DEGs between the DMPtNPS@cGAMP + RT and DMPtNPS + RT groups (Table S3, Supporting Information), 26 upregulated and 79 downregulated (**Figure** **7**A and Figure S23, Supporting Information). GSEA analysis demonstrated cGAS‐STING‐related pathways enrichment, including cytosolic DNA sensing, innate immune activation, and type I interferon responses in the DMPtNPS@cGAMP + RT group as opposed to the DMPtNPS + RT group (Figure 7A). Moreover, in tumors with DMPtNPS@cGAMP + RT group, p‐STING, p‐IRF3, and p‐TBK1 were upregulated compared to other treatment groups. Similarly, the PD‐L1 expression was elevated in DMPtNPS@ cGAMP + RT group (Figure 7B). These findings propose effective delivery of cGAMP to the tumor site with subsequent activation within the tumors.

The cGAS‐STING pathway initiates innate immunity by recognizing cytosolic DNA and generating type I interferons.^[^ ^18^ ^]^ Tumor‐infiltrating natural killer (NK) cells were markedly increased with DMPtNPS@cGAMP + RT group (57.9%) in contrast to RT (18.8%), RT + cGAMP (24.1%), or RT + DMPtNPS groups (41.0%) (Figure 7C). The cGAS‐STING also promotes DC maturation and antitumor immunity.^[^ ^16^, ^17^ ^]^ FCM data demonstrates that treatment with DMPtNPS@cGAMP + RT led to a 1.8‐fold increase in DC1s, a 2‐fold increase in mDCs, and 1.8‐fold increase in tumor‐infiltrating CD3^+^CD8^+^ T cells when compared to the DMPtNPS + RT group (Figure 7D–F). Immunofluorescence showed an increase in IFN‐γ, PD‐L1, CD8^+^T cells, and NK cells in the DMPtNPS@cGAMP + RT group compared to the DMPtNPS + RT group (Figure 7H). We observed a decrease in Tregs within the DMPtNPS@cGAMP + RT group through both FCM and immunofluorescence analysis (Figure 7G,H). This finding suggests that the decrease in Tregs may be a crucial element in DMPtNPS@cGAMP's ability to reverse the negative effects of RT and DMPtNPS on the TiME.

The DMPtNPS@cGAMP + RT group exhibited a significant increase in anti‐tumor cytokines, namely IFN‐γ, IFN‐α, IFN‐β, TNF‐α, and IL‐2, and a more pronounced decrease in pro‐tumor cytokines, including IL‐1β, IL‐10, IL‐4, and TGF‐β, as measured by ELISA kits, in comparison to the other treatment groups (Figure 7I and Figure S24, Supporting Information). The DMPtNPS@cGAMP + RT group exhibited an increase in C‐X‐C motif chemokine ligand 10 (CXCL10), which is accountable for the recruitment of CXCR3+ T cells to the tumor site as well as being a marker of activated CD8+T cells.^[^ ^39^ ^]^ This increase was not observed in the other treatment groups. Although the mechanisms require further clarification, it is hypothesized that administering exogenous cGAMP has a positive impact on the TiME. This effect is achieved through an increase in immune cell infiltration, a reduction in Tregs infiltration, and a decrease in suppressive cytokines (Figure 7J). Ultimately, these changes may lead to improved response to immunotherapy.

### 2.7 αPD‐1 Therapies Enhanced the Antitumor Immune Response of DMPtNPS@cGAMP and RT

**Figure 8:**
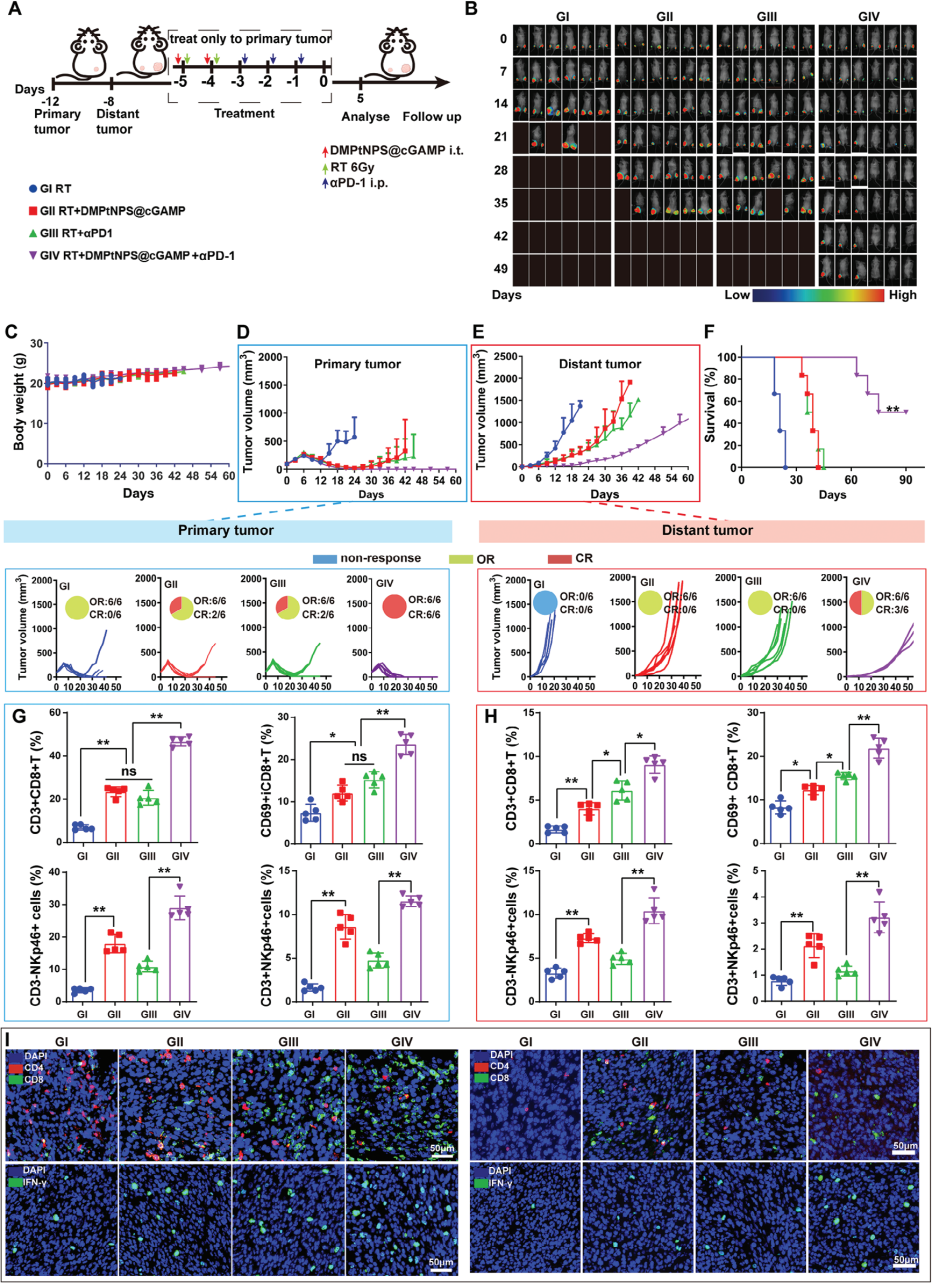
Antitumor effect and TiME modulation of triple therapies. A) The workflow depicts the establishment of the bilateral subcutaneous xenograft tumor model and the triple therapies. B) Bioluminescence imaging of CT26‐luc‐bearing mice every 7 d after receiving different treatments until day 49 (*n* = 6). C) Changes of body weight of CT26‐luc bearing mice in each treatment group. Tumor volume and treatment responses of D) the primary tumor and E) distant tumor (*n* = 6). F) Survival curves of mice after different treatments (*n* = 6). Flow cytometry of CD8^+^T cells, CD8^+^CD69^+^ T cells, CD3^−^NKp46^+^NK cells, and CD3^+^NKp46^+^NKT cells in G) the primary and H) distant tumors after receiving different treatments as indicated (*n* = 5). I) Immunofluorescence images of CD4^+^T cell (red), CD8^+^T cell (green), and IFN‐γ (green) in the primary and distant tumors. The nucleus was stained blue. Scale bar, 50 µm. ns indicates not significant, *P < 0.05, **P < 0.01. Data are presented as mean ± SD.

The above findings demonstrate that upregulation of PD‐L1 and improvement of the TiME by RT and DMPtNPS@cGAMP create a viable combination treatment with anti‐PD1. We established a bilateral tumor mouse model to evaluate the efficacy of triple therapies using RT, DMPtNPS@cGAMP, and αPD‐1 (**Figure** **8**A,B). Throughout the treatments, no significant differences in body weight were observed between the groups (Figure 8C). Compared to single therapies, including RT alone (GI), DMPtNPS@cGAMP + RT (GII), or αPD‐1 + RT (GIII), all six mice (100%) in the group receiving triple therapies (GIV) achieved CR at the primary site, and three mice achieved CR at the distant site (Figure 8D,E). Triple therapy significantly prolonged the median OS of the mice to 82.5 d, in contrast to GI (21 d), GII (37.5 d), and GIII (39 d) groups. Furthermore, two mice (33.3%) in the GIV group were still alive on day 90 (Figure 8F). Additionally, the GIV group exhibited higher levels of tumor necrosis and apoptosis in both primary and distant tumors compared to the other groups, as evidenced by H&E and TUNEL staining analysis (Figure S25, Supporting Information). These results suggest that the use of triple therapies results in potent and long‐lasting anti‐tumor effects, ultimately leading to improved survival.

The enhancement of the TiME may be crucial for the efficacy of triple therapies. We noted a considerable increase in the percentage of CD8^+^T cells infiltrated the primary tumor upon triple therapies (46.7% ± 0.9%), which was significantly higher than the obtained for the GI (7.0% ± 0.6%), GII (20.7% ± 1.5%), or GIII (23.5% ± 1.0%) groups (Figure 8G). In addition, CD69, identified as an early marker of T cell activation, was also observed.^45^ The proportion of CD69^+^CD8^+^ T cells in the primary tumor was considerably higher in the GIV group (23.6% ± 1.1%) compared to the GI (7.4% ± 2.0%), GII (12.1% ± 0.8%), or GIII (15.2% ± 0.9%) (Figure 8G). Therefore, it can be inferred that the triple therapy effectively activates CD8^+^T cells in the primary tumor. NK cells play a crucial role in the innate immune system,^[^ ^40^ ^]^ while NKT cells possess both T cell and NK cell receptors.^[^ ^41^ ^]^ NKT cells can produce various cytokines, similar to T cells, and possess cytotoxic effects like NK cells. The FCM results revealed that triple therapies significantly increased the infiltration of NK and NKT cells into the primary tumor compared to the other groups. Similarly, distant tumors also showed an increased infiltration of CD3^+^CD8^+^T cells, CD69^+^CD8^+^T cells, NK cells, and NKT cells after triple therapies (Figure 8H). In addition, triple therapies resulted in a decrease in CD3^+^CD4^+^T cell levels (Figure S26, Supporting Information). Immunofluorescence analysis indicates increased CD8^+^ staining and reduced CD4^+^ staining in both primary and distant tumors after triple therapies (Figure 8I). Further, IFN‐γ levels were considerably elevated in both primary and distant tumors (Figure S27, Supporting Information). Together, triple therapies activate antitumor immunity, achieving durable CR in primary tumors and an amplified “abscopal effect” in distant tumors.

## 3 Discussion

Catalytic radiosensitizers containing PtNPs have been analyzed to improve hypoxia by catalyzing O_2_ production from H_2_O_2_.^[^ ^13^ ^]^ However, insufficient attention has been paid to the unfavorable side effects of RT on the TiME. In this study, we utilized in situ synthesis to load PtNPs into DM and produce the catalytic radiosensitizer of DMPtNPS, with uniform morphology and stability, the highest PtNP loading, and the capacity to produce O_2_. The in vitro studies showed that DMPtNPS enhances RT‐induced DNA DSB and apoptosis through ROS release (Figure 2K,L). DMPtNPS also mitigates tumor hypoxia while promoting apoptosis and delaying non‐durable responses to RT in vivo (Figure 4F). Furthermore, transcriptomics showed that combining DMPtNPS and RT leads to enhanced DNA damage repair and hypoxia response pathways compared to the RT alone. This observation indicates that DMPtNPS is capable of overcoming the radioresistance of RC by producing O_2_ and releasing ROS in tumors.

However, the limited response to RT and DMPtNPS implied that catalytic radiosensitizers alone were ineffective in achieving durable CR. RT has dual effects on the TiME. On one hand, it can enhance antigen presentation, DC activation, and anti‐tumor CD8^+^T cell responses via ICD.^[^ ^6^, ^24^, ^26^ ^]^ On the other hand, it can also induce immunosuppression through cytokines like TGF‐β,^[^ ^42^ ^]^ which contributes to radioresistance.^[^ ^27^ ^]^ Catalytic radiosensitizers can enhance the bidirectional remodeling effect of RT on the TiME, but cannot reverse the negative side effect of RT. In our study, we found that DMPtNPS augmented RT‐induced ICD and activation of cGAS‐STING pathway, leading to a complex TiME with elevated immune‐suppressive and ‐persuasive cells, and pro‐ and anti‐tumor cytokines. Additional strategies are necessary to disrupt the delicate equilibrium between the favorable and unfavorable impact of DMPtNPS + RT on the TiME. These strategies have the potential to reconfigure a more effective TiME for the maintenance of a prolonged antitumor immune response.

Recent studies have shown that delivering cGAMP via nanoparticles has the potential to reprogram the TiME.^[^ ^33^, ^34^ ^]^ As part of this study, we incorporated cGAMP onto the catalytic radiosensitizer, resulting in DMPtNPS@cGAMP with a drug loading ratio of 12.8 wt%. Surprisingly, 33.3% of the mice achieved durable CR after treatment with DMPtNPS@cGAMP and RT, which was not observed in the groups treated with RT and DMPtNPS or RT alone. Increased levels of DCs, CD8^+^T cells, and NK cells but decreasing Tregs were observed in the DMPtNPS@cGAMP and RT group compared to other groups, along with increased anti‐tumor cytokines and reduced pro‐tumor cytokines. These findings suggest that delivering cGAMP via DMPtNPS can reverse the negative effects of RT and DMPtNPS on the TiME, which was consistent with the previous results from single cell sequencing of glioblastoma treated with B‐LNP/diABZI.^[^ ^20d^ ^]^ Therefore, we determined that DMPtNPS@cGAMP serves as both a catalytic radiosensitizer, enhancing RT‐induced cytotoxicity and an immunomodulator, prompting the reshaping of the TiME for optimal immunotherapy outcome.

iRT has garnered significant attention in recent years. As a local treatment, RT not only ensures satisfactory local control but also initiates an antitumor immune response through numerous pathways. As a systemic treatment, immunotherapy provides a systemic effect and long‐term benefits. In this study, the combination of RT and DMPtNPS@cGAMP resulted in improved TiME and upregulation of PD‐L1, which provided a rational regimen for subsequent anti‐PD1 treatment. The achievement of durable CR in all primary tumors (100%) using triple therapies of RT, αPD‐1 and DMPtNPS@cGAMP is a promising result, as it is accompanied by a prolonged median OS (Figure 8D,F). Further analysis showed that the triple therapies further improved the TiME by enhancing the function of CD8+T cells, NK, and NKT cells, which may be the key to the durable CR.

In addition, achieving an “abscopal effect” is a desired outcome of immunoradiotherapy.^[^ ^6^, ^43^ ^]^ The incidence of the “abscopal effect” has historically been low, and reported only in case reports.^[^ ^6^, ^8^ ^]^ In a phase II study, 24 metastatic mismatch repair‐competent patients, who are much more sensitive to RT and ICIs, received RT and two ICIs (durvalumab plus tremelimumab). However, only 8.3% of patients achieved partial response in the unirradiated sites.^[^ ^46^ ^]^ The limited response observed may contribute to the immunosuppressive TiME. Our bilateral tumor mouse model demonstrated the triple therapies achieved a durable CR in 50% of distant tumors (Figure 8E). And two mice (33.3%) that received triple therapies remained alive until day 90 (Figure 8F). Further analysis revealed an improvement in the TiME of distant tumors treated with the triple therapies, indicated by increased CD8+T cells, NK, and NKT cells. This amplified “abscopal‐effect” was consistent with previous findings using cGAMP delivery through tannic acid nanostructure^[^ ^45^ ^]^ or hafnium‐based nanoparticles,^[^ ^46^ ^]^ emphasizing the feasibility of triple therapies in inducing systemic antitumor immunity and the desired “abscopal effect.”

In conclusion, our study showed that the DMPtNPS@cGAMP, a catalytic radiosensitizer, enhances the efficacy of RT treatment by remodeling the TiME and boosting antitumor immunity. Furthermore, the combination of DMPtNPS@cGAMP, RT, and ICIs has shown the potential to induce an amplified “abscopal effect.” This novel treatment offers new possibilities for treating RC. This study highlights the potential of merging cGAMP with catalytic radiosensitizers in reconfiguring the TiME, amplifying anti‐tumor immune responses, and furnishing an advantageous accessory to cancer therapy that may result in enhanced therapeutic outcomes in RC patients.

## 4 Experimental Section

### Materials

Tetraethylorthosilicate (TEOS), 3‐aminopropyltrimethoxysilane (APTES), DAPI, and 2, 7‐dichlorodihydrofluorescein diacetate (DCFH‐DA) were purchased from Sigma‐Aldrich. Hexadecyl trimethylammonium chloride (CTAC), triethanolamine (TEA), sodium borohydride (NaBH_4_), and chloroplatinic acid (H_2_PtCl_6_) were purchased from Sinopharm Chemical Reagent Co. Ltd. CalceinAM/ propidium iodide (PI) was purchased from KeyGen BioTech (Shanghai, China). Annexin V‐FITC/propidium iodide (PI) and Cell Counting Kit 8 (CCK8) kit were obtained from Dojindo Laboratories. αPD1 was purchased from BioLegend, Inc (USA). cGAMP and its fluorescent analogue were purchased from BIOLOG Life Science Institute (Germany).

### Preparation and Characterization of DMPtNPS@cGAMP

DM was synthesized using previously reported methods with CTAC and TEOS.^[^ ^21b^ ^]^ To optimize the PtNPs loading on DMPtNPS, H_2_PtCl_6_·6H_2_O was added in varying ratios to NaBH_4_ (5:1, 10:1, 20:1) for the in situ loading of PtNPs. cGAMP was mixed with DMPtNPS at a ratio of 1:5 ratio in 5 × 10^−3^ m histidine buffer under ultrasound for 30 min for cGAMP loading.

Nanoparticle morphology was observed by TEM using an FEI Tecnai instrument (FEI Company, Hillsboro, OR). Dynamic light scattering was used to measure particle size and zeta potential using a Malvern Zetasizer Nano ZS (ZEN3600, UK). UV–vis absorbance was measured using a SpectraMax M5 microplate reader (Germany). The oxidation state of the nanoparticles was analyzed by X‐ray photoelectron spectroscopy using a Thermo Scientific Escalab 250Xi instrument. Elemental analysis was conducted using an Agilent ICPMS7800 inductively coupled plasma mass spectrometer.

### Cell Culture and Animals

CT26 cells (murine CRC cells), HCT116 cells (human colorectal cancer cells), DC2.4 cells (mouse dendritic cell line ), and HUVEC cells (human umbilical vein endothelial cells) were acquired from ATCC. These cells were cultured in Roswell Park Memorial Institute (RPMI) 1640 medium at 37 °C in a humidified atmosphere with 5% CO_2_. The RPMI‐1640 medium was supplemented with 10% fetal bovine serum (FBS) and 1% penicillin–streptomycin.

For the purpose of in vivo studies, BALB/C mice (female, 6–8 weeks old) were purchased from the Shanghai Wushi Experimental Animal Center (Shanghai, China). All animal studies were approved by the Institutional Animal Care and Use Committee at Fujian Medical University.

### Cellular Uptake of DMPtNPS

To evaluate the cellular uptake of DMPtNPS, immunofluorescence and FCM were employed. For immunofluorescence analysis, 1 × 10^5^ CT26 and HCT116 cells were seeded in 20 mm confocal dishes for 24 h. Afterward, the cells were then coincubated with Cy5‐labeled DMPtNPS (40 µg mL^−1^) at different time points (0, 4, 8, and 24 h). Subsequently, the treated cells were fixed with 4% paraformaldehyde and stained with DAPI (1 mg mL^−1^, Dojindo Molecular Technologies) for 15 min. Afterwards, the treated cells were visualized by CLSM (LSM 780, Germany) and their quantified was measured using FCM (BD Biosciences) with gating strategy in Figure S28A (Supporting Information).

### Cell Viability Assay and Cytotoxicity of DNPtNPS

Cell viability was assessed using the CCK8 assay (MedChemExpress, Shanghai, China). A total of 8 × 10^3^ cells per well of CT26, HCT116, DC2.4, and HUVEC cells were seeded in 96‐well plates and allowed to adhere for 24 h before treatment. The cells were then treated with different concentrations of DNPtNPS (0, 1.25, 2.5, 5, 10, 20, 40, 60, 80, and 100 µg mL^−1^). After incubation for 6 h, the cells were exposed to X‐ray radiation (6 Gy). After 48 h of coincubation, cell viability was assessed using the CCK8 assay. The absorbance of each well was measured at 450 nm using the microplate reader (Spectra Max M5, Germany). Cell viability was determined using the CCK8 method based on the manufacturer's instructions. The antitumor efficacy of each group was also evaluated using live/dead viability/cytotoxicity kit staining (Calcein‐AM). Additionally, the proliferation of cells was assessed via a colony formation assay subsequent to treatment with DMPtNPS combined with RT, in comparison with the DM, DMPtNPS, RT, and RT + DM groups. In addition, the Annexin V apoptosis assay kit (MedChem Express, Shanghai, China) was employed to determine cell apoptosis in each group after treatment, using FCM with gating strategy in Figure S28B (Supporting Information).

### Intracellular Reactive Oxygen Species (ROS) Level Detection

To detect intracellular levels of ROS, the DCFH‐DA fluorogenic probe was utilized (Sigma‐Aldrich, USA).^[^ ^47^ ^]^ CT26 and HCT116 cells were seeded in 96‐well plates at a concentration of 1 × 10^4^ cells per well and coincubated with DNPtNPS (40 µg mL^−1^) for 6 h. After staining with 50 × 10^−6^ m DCFH‐DA for 30 min, the treated cells were washed with PBS buffer prior to exposure to 6 Gy X‐ray irradiation. After exposure to 6 Gy of X‐ray irradiation, the cells were viewed through a Nikon fluorescence microscope with excitation at 488 nm.

### DNA Damage Evaluation

To detect DNA damage, γ‐H2AX was utilized, a marker of DNA DSB.^[^ ^48^ ^]^ Immunofluorescence analysis and FCM were used for analysis. CT26 and HCT116 cells (1 × 10^5^) were seeded into 20 mm confocal dishes for immunofluorescence and 12‐well plates for FCM with the gating strategy in Figure S28C (Supporting Information). Following treatment with DNPtNPS (40 µg mL^−1^) and 6 Gy X‐ray radiations, cells were fixed in 4% paraformaldehyde and blocked with a 0.1% Triton solution. The cells were then incubated overnight at 4 °C with a primary antibody against γ‐H2AX (ab22551, Abcam, 1:100). Then, cells were incubated with a secondary antibody conjugated to Alexa Fluor 647 (ab150115, Abcam, 1:500) for 2 h, followed by staining of cells plated in confocal dishes with DAPI. Imaging was conducted using CLSM, while analysis of cells plated in 12‐well plates was carried out using FCM. Data analysis was performed utilizing FlowJo software.

### Immunogenic Cell Death (ICD) Effect of DNPtNPS

To assess ICD, the exposure to CRT, HMGB‐1 exposure, and ATP release were examined.^[^ ^24^ ^]^ Immunofluorescence analysis and FCM were employed to identify surface‐exposed CRT. For immunofluorescence analysis, CT26 and HCT116 cells (1 × 10^5^) were seeded in 20 mm confocal dishes, while FCM analysis was carried out in 12‐well plates. After treatment with DNPtNPS (40 µg mL^−1^) and 6 Gy of X‐ray radiations, the cells were fixed in 4% paraformaldehyde. Next, they were incubated with a primary antibody against CRT (ab92516, Abcam, 1:100) for one night at 4 °C. This was followed by incubation with a secondary antibody conjugated to Alexa Fluor 647 (ab150115, Abcam, 1:500) for 2 h. FCM analysis was carried out with gating strategy in Figure S28D (Supporting Information), and CLSM was used to visualize the cells after incubation with DAPI.

Extracellular HMGB1 levels were quantified using an HMGB‐1 ELISA kit (Fankewei, Shanghai, China). Intracellular and extracellular ATP levels were measured using an ATP assay kit (Beyotime, Shanghai, China). CT26 and HCT116 cells were seeded in six‐well plates at a density of 1 × 10^6^ cells per well, and were subsequently subjected to DNPtNPS (40 µg mL^−1^) and 6 Gy of X‐ray radiation. After treatment, the supernatants were evaluated utilizing the HMGB‐1 ELISA kit, as per the manufacturer's instructions. Intracellular and extracellular levels of ATP were quantified utilizing the ATP assay kit following the protocol suggested by the manufacturer.

### Maturation of Bone‐Marrow‐Derived Dendritic Cells (BMDC) and T Cell Activated

BMDCs were extracted from the bone marrow of BALB/c mice and grown in 24‐well plates with RPMI‐1640 medium containing 10% FBS, 1% penicillin‐streptomycin, GM‐CSF (20 ng mL^−1^, MedChemExpress, Shanghai, China), and IL‐4 (10 ng mL^−1^, MedChemExpress, Shanghai, China).^[^ ^49^ ^]^ CT26 cells were seeded at a concentration of 1 × 10^5^ per well in the upper compartment of transwell inserts. Various treatments were then administered to the tumor cells. On day 6, the tumor cells were incubated with BMDCs and placed in the lower chamber for 24 h. Following co‐incubation, the BMDCs were harvested and stained using the following antibodies: Anti‐CD8‐FITC (53‐6‐7, BioLegend), Anti‐CD11C‐APC (17‐0114‐82, eBioscience), Anti‐CD80‐PE (12‐0801‐82, eBioscience), and Anti‐CD86‐PE/Cyanine7 (PO3, BioLegend). All samples were analyzed via FCM with gating strategy in Figure S28E (Supporting Information). Furthermore, the ELISA kits (Fankewei, Shanghai, China) were utilized to collect and examine the supernatants of BMDCs for TNF‐α, IL‐12, IL‐10, and TGF‐β, following the manufacturer's instructions.

T cells were obtained by isolating splenocytes from BALB/C mice and subjecting them to Ficoll separation (P4350, Solarbio). The T cells were then placed on antibody‐coated plates at a density of 2 × 10^5^ cells per well. BMDCs were cocultured with tumor cells. Following this, the T cells were coincubated with BMDCs at 37 °C for 48 h. The manufacturer's instructions were followed to process the antibody‐coated plate, which was then imaged using an ImmunoSpot Analyzer (Cellular Technology Ltd., US).

### CT26 Tumor‐Bearing Model Established

The study utilized unilateral and bilateral tumor models with CT26‐luc cells. To establish the unilateral model, 5 × 10^5^ CT26 cells were subcutaneously injected into the right hind leg of Balb/C mice, defining it as the primary tumor. The UniNano NIR‐II imaging system confirmed the established tumor model after injecting luciferin. For the bilateral tumor model, 2 × 10^5^ CT26‐luc was injected into the secondary tumor 3 days after implanting the primary tumor. Tumor‐bearing mice were randomly allocated to groups when the primary tumor size reached approximately 100 mm^3^.

### Combined Therapeutic Efficiency Assessment In Vivo

The external caliper was utilized to measure the longitudinal diameter (*L*) and transverse diameter (*W*). Formula tumor volume (mm^3^) = [*L*(mm) × *W*(mm)^2^] / 2^[^ ^50^ ^]^ was employed to calculate the tumor volume. Before randomization and treatment, mice were weighed and tumor volume was measured. One group of mice (*n* = 5) from each group was sacrificed 5 d after treatment for further analysis, while the remaining mice (*n* = 6) were observed for survival. Tumor volume and mouse weight were measured every 3 d. Bioluminescent images were obtained weekly using the UniNano NIR‐II real‐time in vivo imaging system, following intraperitoneal injection of D‐luciferin (15 mg mL^−1^, 200 µL per mouse) (122799, PerkinElmer). The tumors were stained with H&E and underwent TUNEL to assess treatment efficacy and changes in tumor pathology resulting from various treatments.

### Immunoassay of DNPtNPS or cGAMP@DNPtNPS in TiME

To obtain the tumor‐infiltrating lymphocytes, fresh tumor tissue was collected 5 d after treatment. To analyze DC maturation, the following antibodies were used for staining: Anti‐CD11c‐APC (17‐0114‐82, eBioscience) antibodies, anti‐CD8a‐FITC (12‐0801‐82, eBioscience), anti‐CD80‐PE (12‐0801‐82, eBioscience), and anti‐CD86‐Cy7 (25‐0862‐82, eBioscience), with gating strategy in Figure S29A (Supporting Information). For the analysis of Treg infiltration, the following antibodies were used for staining: Anti‐CD4‐FITC (11‐0042‐85, eBioscience), anti‐CD25‐per‐Cy5.5 (45‐0251‐82, eBioscience), and anti‐Foxp3‐PE‐Cy7 (25‐5773‐82, eBioscience), with gating strategy in Figure S29B (Supporting Information). For the analysis of effective CD8+T cell infiltration, the following antibodies were used for staining: Anti‐CD3‐APC (17‐0032‐82, eBioscience), anti‐CD8‐PE (12‐0081082, eBioscience), and anti‐CD69‐FITC (H1‐2F3, BioLegend), with gating strategy in Figure S29C (Supporting Information). For the NK cells, the following antibodies were used for staining: Anti‐CD3‐APC (17‐0032‐82, eBioscience), and NKp46‐PE/Cyanine7 (25‐4321‐82, eBioscience), with gating strategy in Figure S29D (Supporting Information).

Cytokines in tumor tissue samples were analyzed using ELISA kits supplied by Fankewei (Shanghai, China), including kits for IFN‐γ, IFN‐α, IFN‐β, IL‐12, IL‐1β, IL‐10, IL‐4, and TGF‐β. The analysis was executed under the manufacturer's instructions. Samples of tumor tissues obtained from mice were collected for immunofluorescence analysis. The cited staining antibodies are as follows: anti‐γ‐H2AX (ab81299, Abcam, 1:200), anti‐CRT (ab92516, Abcam, 1:100), anti‐CD4 (bs‐0766R, Bioss, 1:200), anti‐CD8 (bs‐10699R, Bioss, 1:200), anti‐Foxp3, anti‐IFN‐γ (bs‐0480R, Bioss, 1:200), and anti‐HIF1‐α (bs‐0737R, Bioss).

### Western Blot Assay

Tumor tissue and cell lysates were prepared using RIPA lysis buffer from Beyotime Biotechnology in China, along with PMSF, protease inhibitor cocktail, and phosphatase inhibitors from MedChemExpress. Protein quantification for tumor tissues and cells was performed with the BCA kit from TransGen Biotech in China. Following gel electrophoresis, the proteins were transferred to nitrocellulose membranes. The membranes were blocked with 5% BSA for 1 h at room temperature. Afterward, they were incubated overnight at 4 °C with primary antibodies (Table S4, Supporting Information), followed by incubation with HRP‐conjugated secondary antibodies (ab205718 and ab205719, Abcam, 1:5000) in accordance with the manufacturer's protocol.

### Immunofluorescence Staining

Tumor tissues were dissected using sterile, fixed with 4% paraformaldehyde, embedded, sectioned, and deparaffinized. Subsequently, primary antibodies (Table S5, Supporting Information) were added to tissue sections and incubated overnight at 4 °C. Next, they were treated with secondary antibodies for 20 min at room temperature. Finally, the stained tissue sections were observed using CLSM.

### Bioinformatics Analysis

Tumor tissues of mice (*n* = 4) from each group were collected 5 days after treatment for high throughput RNA sequencing (Table S2, Supporting Information). DEGs between each group were identified (|log_2_FC| ≥ 1, FDR < 0.05). Gene set GSEA was performed using GSEA software (http://software.broadinstitute.org/gsea). Results were visualized using R studio. The data of the GSE56699, GSE233517, and GSE87211 are available at https://www.ncbi.nlm.nih.gov/geo/.

### Statistical Analysis

All data were analyzed using one‐way analysis of variance (ANOVA) to compare multiple groups or two‐tailed Student's t‐tests between two groups using GraphPad Prism 8.0 software. The mouse was euthanized once the tumor volume reached 2000 mm^3^, regardless of primary or distant. Survival curves were constructed using the Kaplan‐Meier method of estimation and tested using the log‐rank test. *P < 0.05, **P < 0.01, ***P < 0.001. *P* values < 0.05 were considered to be statistically significant. All numerical data are presented as the mean ± SD of at least three experiments.

### Ethical Standards

All animal experiments were conducted in accordance with ethical policies and procedures approved by the Animal Care and Use Committee of Fujian Medical University (Approval number: 2021‐3CAARM024).

## Conflict of Interest

The authors declare no conflict of interest.

## Supporting information

Supporting Information

Supplemental Table 1

Supplemental Table 2

Supplemental Table 3

Supplemental Table 4

Supplemental Table 5

## Data Availability

The data that support the findings of this study are available from the corresponding author upon reasonable request.
