# Supplementary material for: STING Agonist‐Loaded Nanoparticles Promotes Positive Regulation of Type I Interferon‐Dependent Radioimmunotherapy in Rectal Cancer
Source: Adv Sci (Weinh). 2023 Dec 8;11(7):2307858. doi: 10.1002/advs.202307858 (PMC10870073; doi:10.1002/advs.202307858)
Supplement: Supplementary file 1 — Supporting Information [file ADVS-11-2307858-s005.pdf]

ADVANCED SCIENCE
Open Access

Supporting Information

for *Adv. Sci.*, DOI 10.1002/advs.202307858

STING Agonist-Loaded Nanoparticles Promotes Positive Regulation of Type I Interferon-Dependent Radioimmunotherapy in Rectal Cancer

Lei Wang, Han Zhou, Qingjing Chen, Zhiwen Lin, Chenwei Jiang, Xingte Chen, Mingdong Chen, Libin Liu, Lingdong Shao, Xiaolong Liu, Jianji Pan, Jingcheng Wu, Jibin Song, Junxin Wu and Da Zhang**

Supporting Information

STING Agonist-Loaded Nanoparticles Promotes Positive Regulation of Type I Interferon-Dependent Radio-Immunotherapy in Rectal Cancer

Lei Wang[1, 2#], Han Zhou[3#], Qingjing Chen[4, 6#], Zhiwen Lin[4, 6], Chenwei Jiang[7], Xingte Chen[1], Mingdong Chen[8], Libin Liu[1], Lingdong Shao[1], Xiaolong Liu[4, 9, 10], Jianji Pan[1], Jingcheng Wu[11], Jibin Song[5], Junxin Wu[1*], Da Zhang[4, 10*]

1. Department of Radiation Oncology, Fujian Cancer Hospital, Fujian Medical University, Fuzhou 350025, P. R. China
2. Department of Oncology, the Second Affiliated Hospital of Nanchang University, Nanchang 360000, P. R. China
3. Department of Clinical Oncology, The University of Hong Kong-Shenzhen Hospital, Shenzhen, Guangdong 518053, P. R. China
4. The United Innovation of Mengchao Hepatobiliary Technology Key Laboratory of Fujian Province, Mengchao Hepatobiliary Hospital of Fujian Medical University, Fuzhou 350025, P. R. China
5. State Key Laboratory of Chemical Resource Engineering, College of Chemistry, Beijing University of Chemical Technology, Beijing 10010, P. R. China
6. Department of Hepatopancreatobiliary Surgery, First Affiliated Hospital of Fujian Medical University, Fuzhou 350004, P. R. China
7. School of Biomedical Engineering, Shanghai Jiao Tong University, Shanghai, 200030, P. R. China
8. Department of Radiation Oncology, Mengchao Hepatobiliary Hospital of Fujian Medical University, Fuzhou 350025, P. R. China
9. CAS Key Laboratory of Design and Assembly of Functional Nanostructures, Fujian Institute of Research on the Structure of Matter, Chinese Academy of Sciences, Fuzhou 350002, P. R. China
10. Mengchao Med-X Center, Fuzhou University, Fuzhou 350116, P. R. China
11. Department of Health Science, Technology and Education, National Health Commission of the People's Republic of China, Beijing 100088, China

[#]L. W, H. Z and Q. C contributed equally to this works
[*]Correspondence: Junxin Wu, Department of Radiation Oncology, Fujian Cancer Hospital, Fujian Medical University, Fuzhou 350025, P. R. China
E-mail: junxinwufj@aliyun.com

*Correspondence: Da Zhang, The United Innovation of Mengchao Hepatobiliary Technology Key Laboratory of Fujian Province, Mengchao Hepatobiliary Hospital of Fujian Medical University, Fuzhou 350025, P. R. China
E-mail: zdluoman1987@163.com;

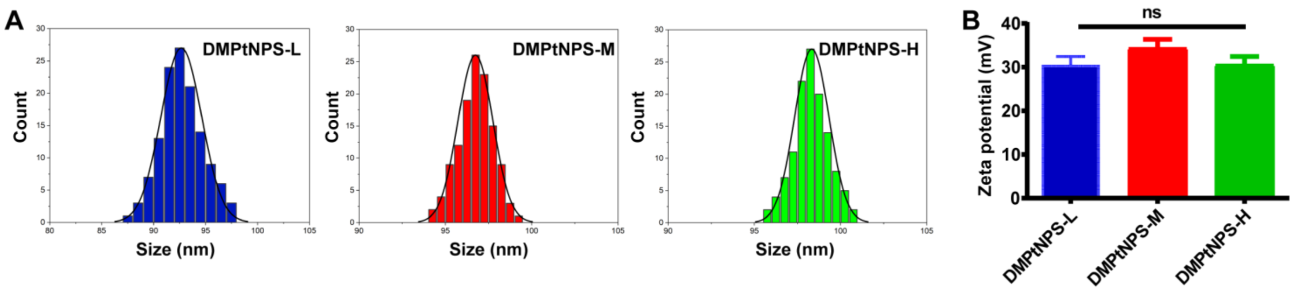

Figure S1. (A) The particle size of DMPtNPS-L, DMPtNPS-M and DMPtNPS-H. (B) The zeta potential of DMPtNPS-L, DMPtNPS-M and DMPtNPS-H. Data are presented as mean ± SD, n=3. Statistical significance was calculated by one-way ANOVA with Tukey's post hoc test. ns indicates no statistical difference.

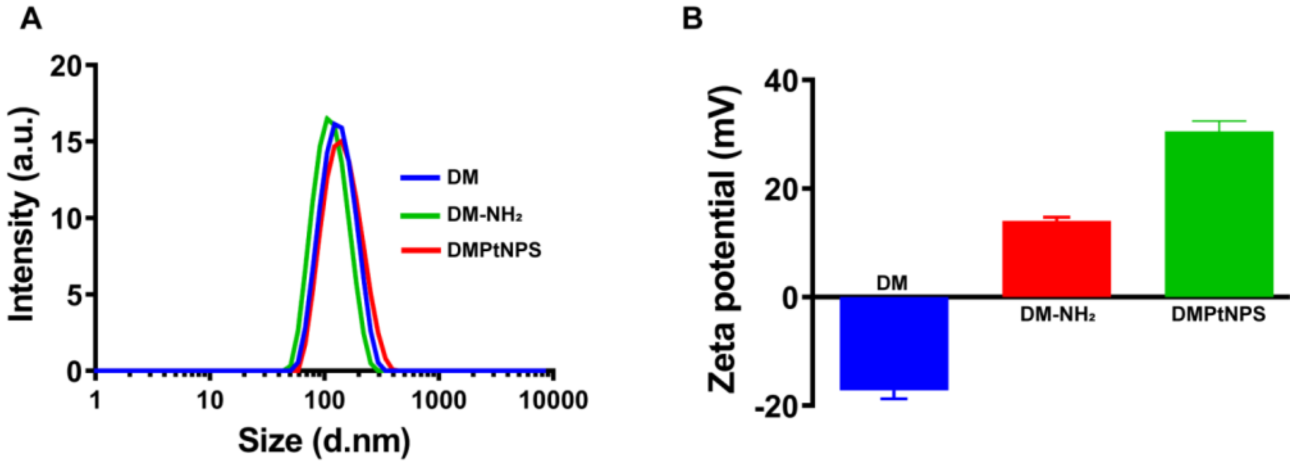

Figure S2. (A) The particle size of DM, $DM\text{-}NH_2$ and DMPtNPS. (B) The zeta potential of DM, $DM\text{-}NH_2$ and DMPtNPS. Data are presented as mean ± SD, n =3.

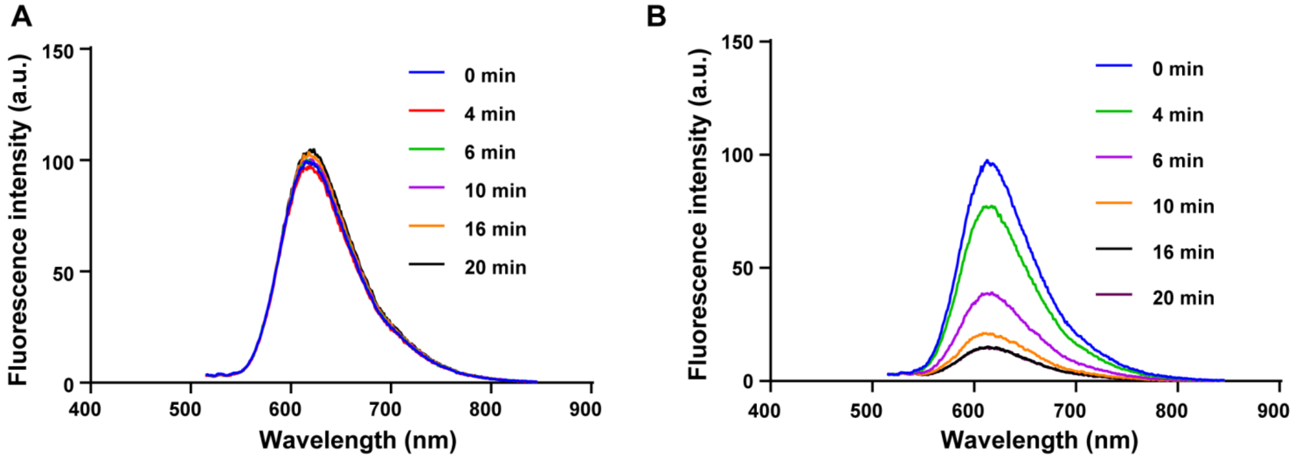

Figure S3. The fluorescence intensity of $Ru(dpp)_3Cl_2$ in DM (A) and DMPtNPS (B) with H_2O_2 (100 μM) for different co-incubation time.

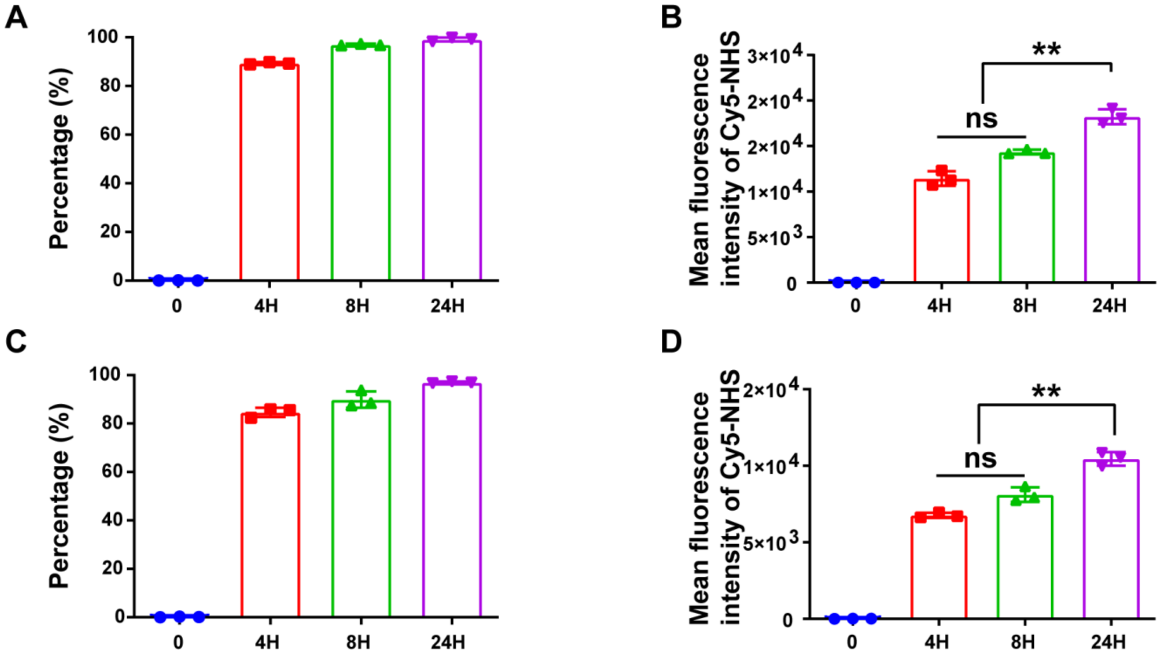

Figure S4. Quantitative flow cytometry of Cy5-NHS labeled DMPtNPS uptake by CT26 cells (A) and HCT116 cells (C). The mean fluorescence intensity of Cy5-NHS labeled DMPtNPS uptake by CT26 cells (B) and HCT116 cells (D). Data are presented as mean ± SD, n = 3. Statistical significance was calculated by one-way ANOVA with Tukey's post hoc test. ns indicates no statistical difference, $^{**}P < 0.01$.

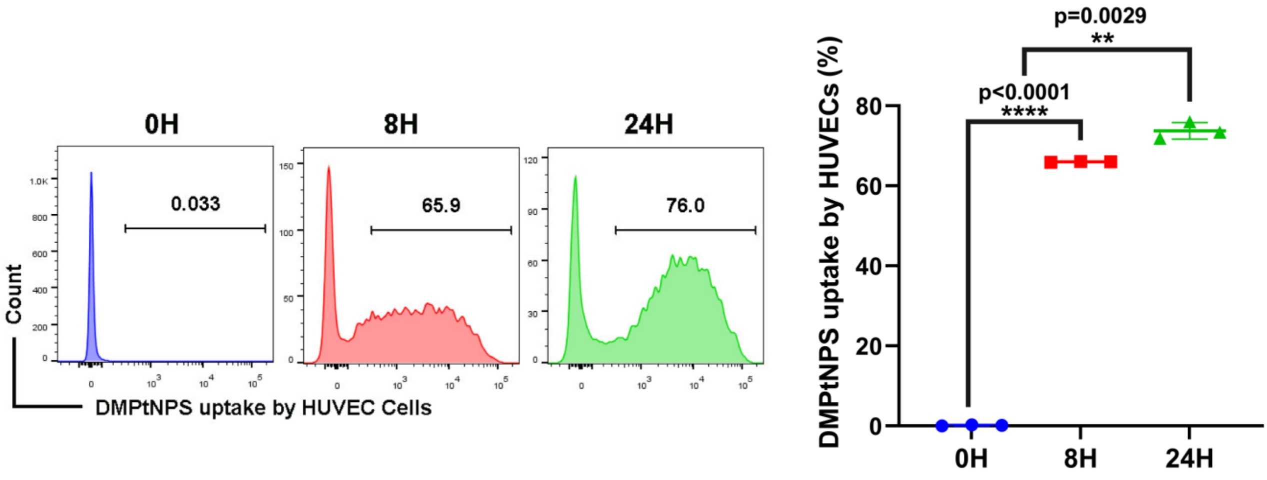

Figure S5. FCM analysis and quantitative analysis of the Cy5-NHS labeled DMPtNPS at 0h, 8h, and 24h after being incubated with HUVEC cells *in vitro*. Data are presented as mean ± SD, n = 3. Statistical significance was calculated by one-way ANOVA with Tukey's post hoc test, $^{**}P < 0.01$, $^{****}P < 0.0001$.

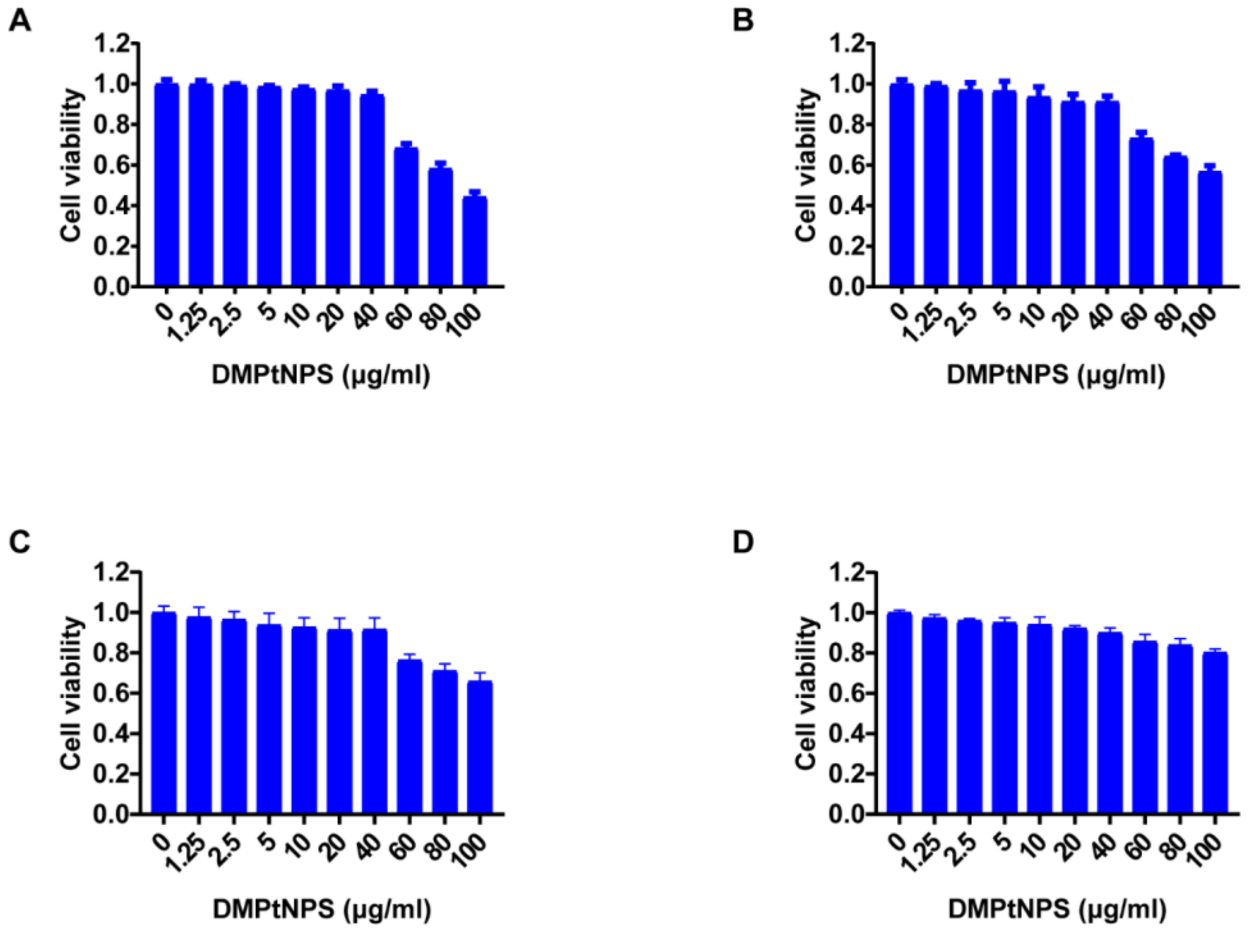

Figure S6. The cell viability of CT26 cells (A), HCT116 cells (B), HUVEC cells (C) and DC2.4 cells after co-incubated with different concentrations of DMPtNPS without X-ray irradiation. Data are presented as mean ± SD, n = 6.

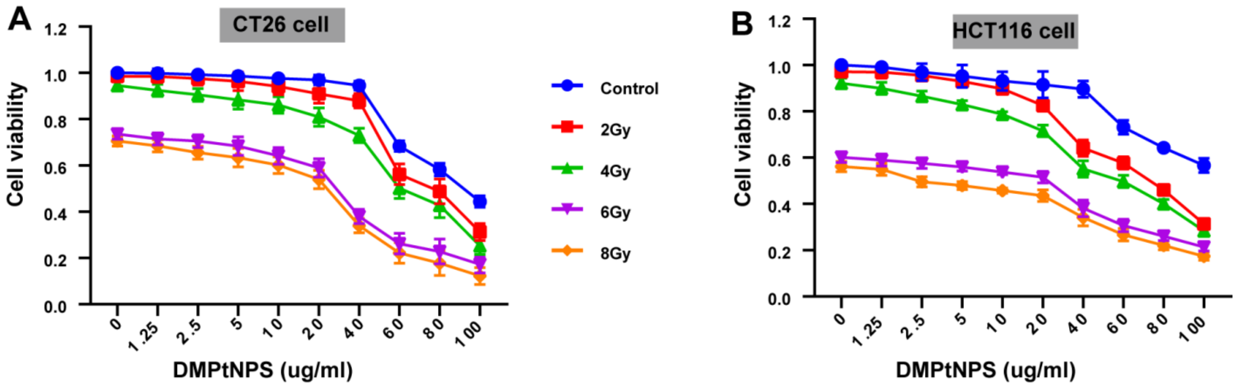

Figure S7. The optimal dose of X-ray and DMPtNPS. The cell viability of CT26 cells (A) and HCT116 cells (B) after treatment with different X-ray dose and at different concentration of DMPtNPS. Data are presented as mean ± SD, n = 6.

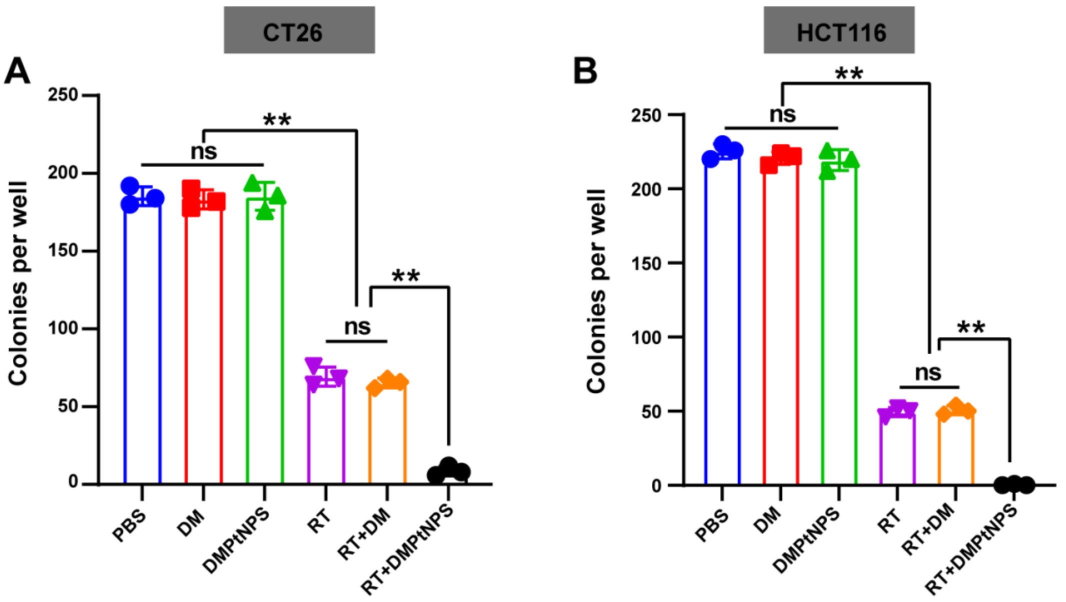

Figure S8. Colony formation quantification of CT26 cells (A) and HCT116 cells (B) after indicated treatments. Data are presented as mean ± SD, n=3. Statistical significance was calculated by one-way ANOVA with Tukey's post hoc test. ns indicates no statistical difference, $^{**}P < 0.01$.

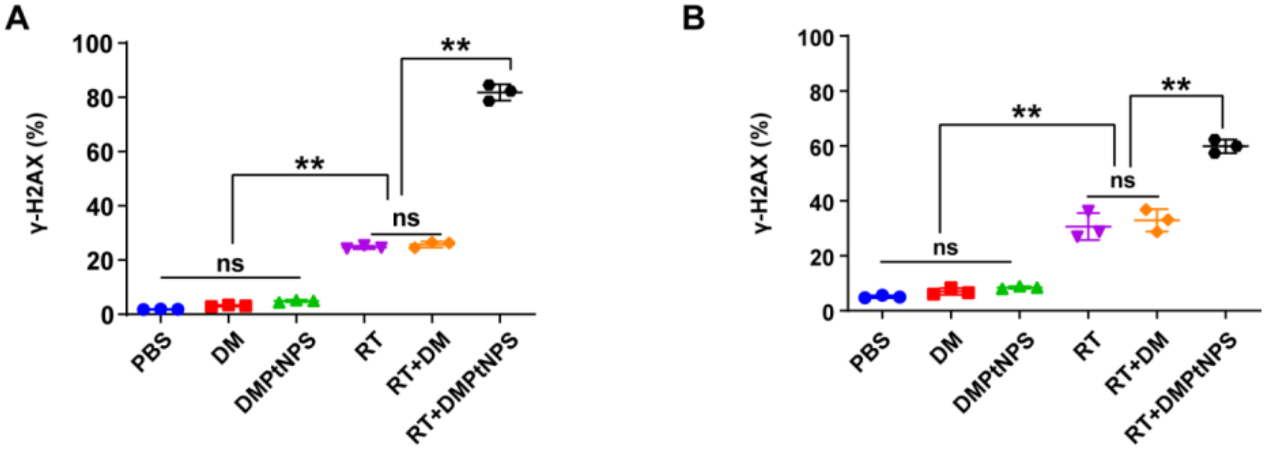

Figure S9. Quantitative analysis of γ-H2AX in CT26 cells (A) and HCT116 cells (B) after indicated treatments. Data are presented as mean ± SD, n=3. Statistical significance was calculated by one-way ANOVA with Tukey's post hoc test. ns indicates no statistical difference, $^{**}P < 0.01$.

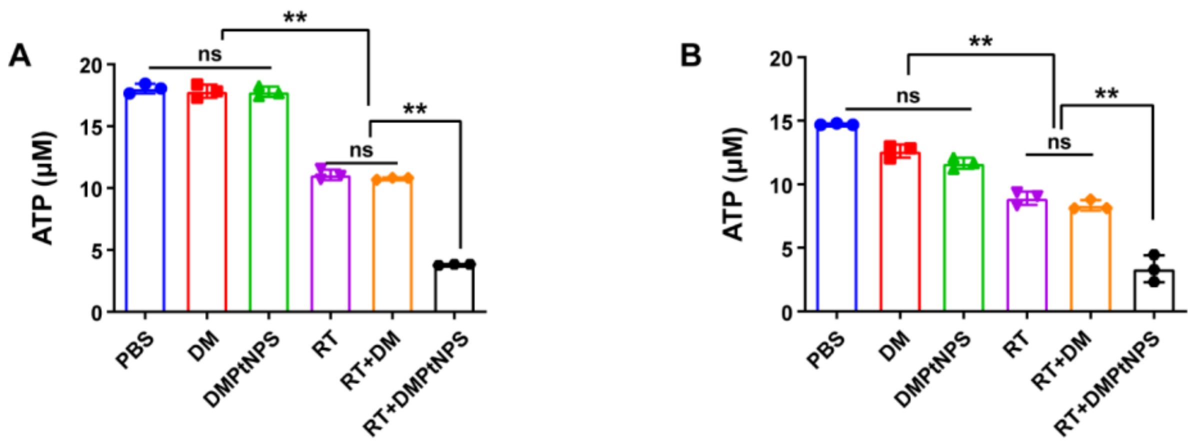

Figure S10. The levels of cytoplasic ATP in CT26 cells (A) and HCT116 cells (B) after indicated treatments. Data are presented as mean ± SD, n=3. Statistical significance was calculated by one-way ANOVA with Tukey's post hoc test. ns indicates n o statistical difference. $^{**}P < 0.01$.

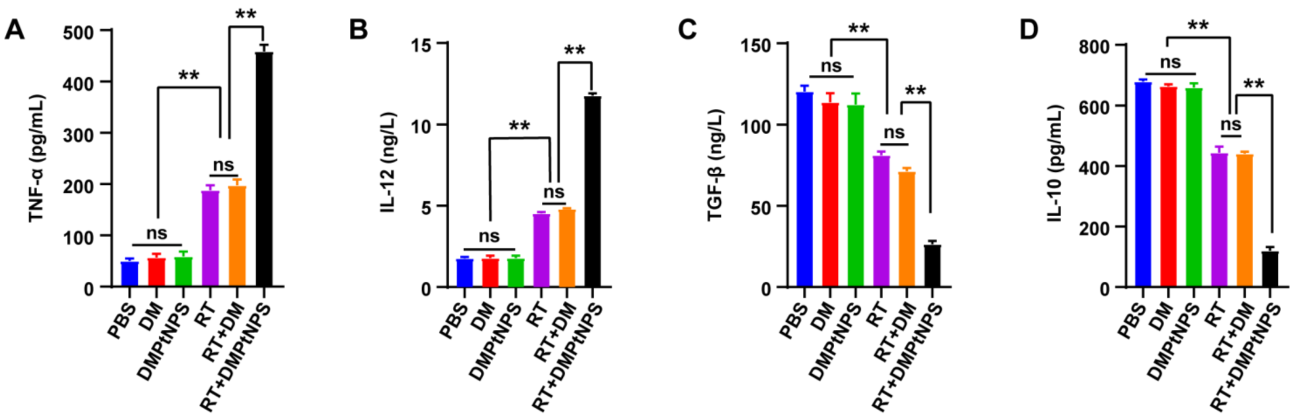

Figure S11. Cytokines levels of TNF-α (A), IL-12 (B), TGF-β (C) and IL-10 (D) from DCs after indicated treatments. Data are presented as mean ± SD, n=3. Statistical significance was calculated by one-way ANOVA with Tukey's post hoc test. Ns indicates no statistical difference, $^{**}P < 0.01$.

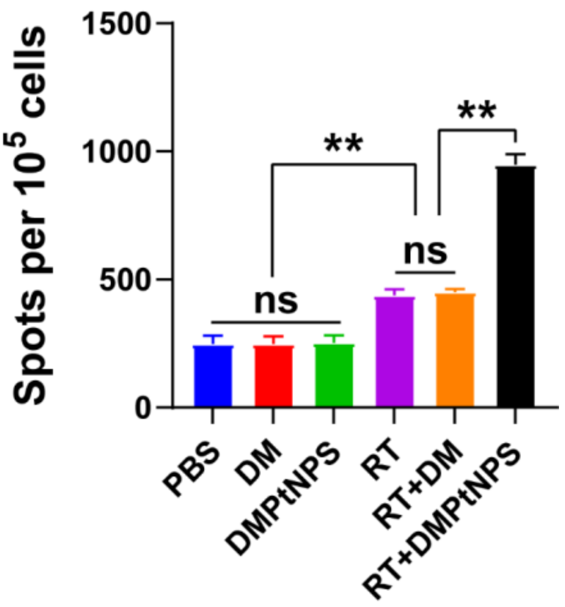

Figure S12. Quantitative analysis of IFN-γ ELISpot after indicated treatments. Data are presented as mean ± SD, n=4. Statistical significance was calculated by one-way ANOVA with Tukey's post hoc test. ns indicates no statistical difference, $^{**}P < 0.01$.

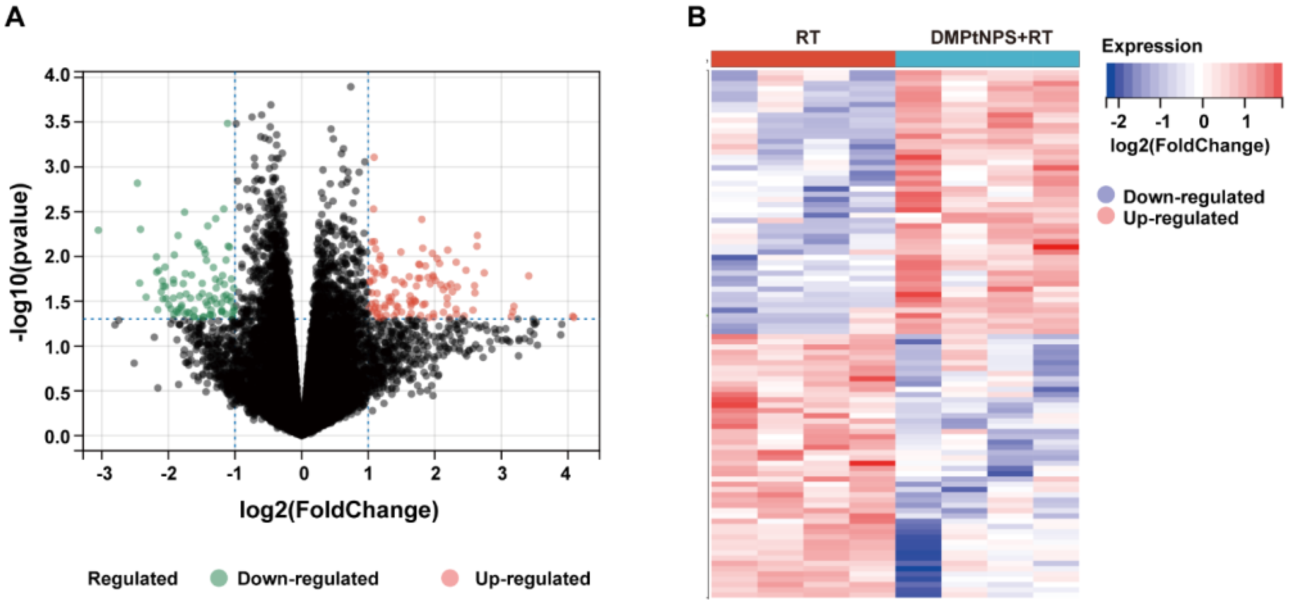

Figure S13. The differentially expressed genes visualised by volcano plot and heat map between RT and DMPtNPS+RT group (n = 4).

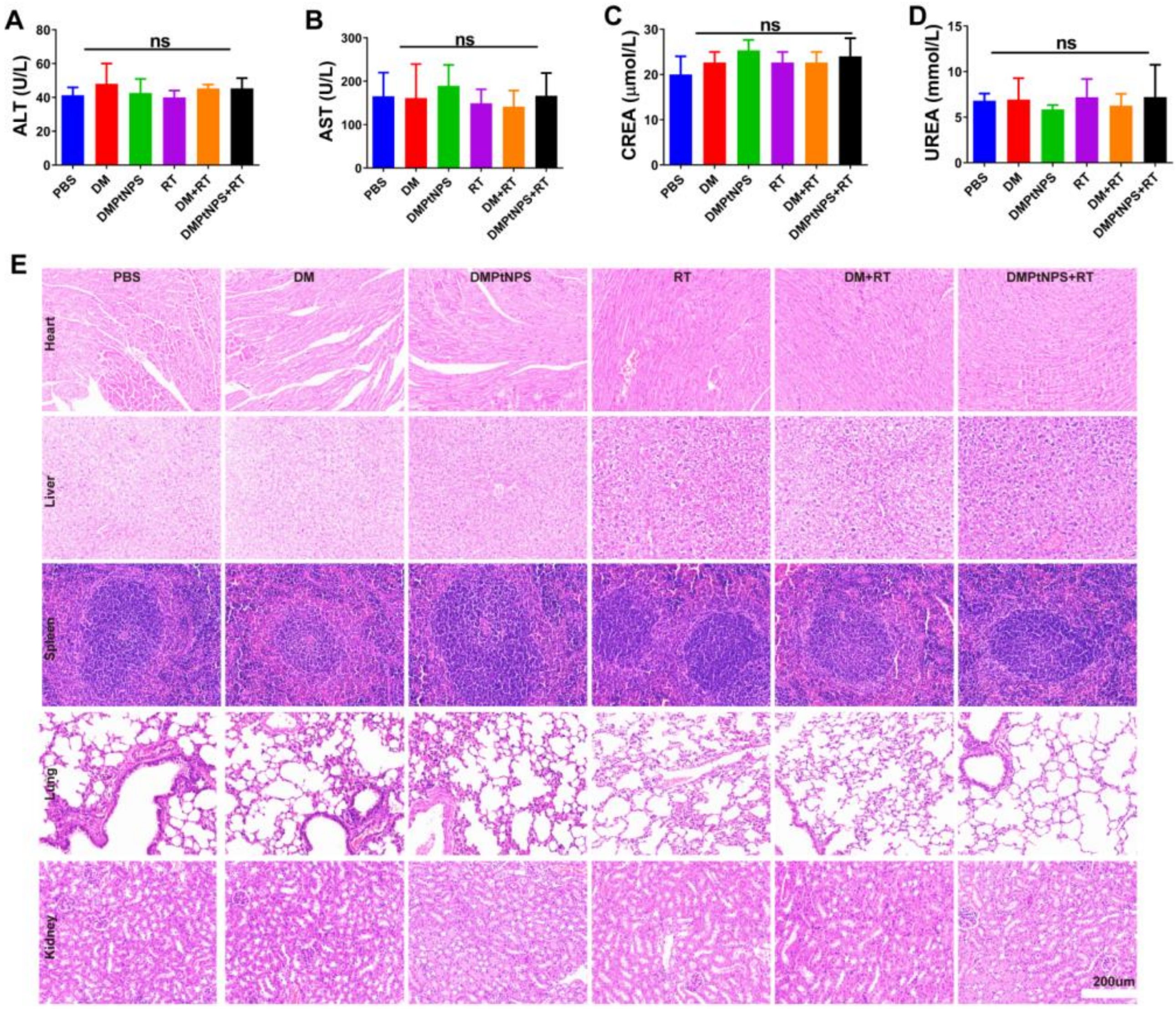

Figure S14. Bio-safety of DMPtNPS combined with RT. Blood biochemistry of alanine aminotransferase (A), aspartate transaminase (B), creatinine (C) and urea (D) after indicated treatments at day 5th. (E) Representative H&E images of major organs (heart, lung, liver, spleen, and kidney) after indicated treatments at day 5th. Scale bar: 200 μm. Data are presented as mean ± SD, n=5. Statistical significance was calculated by one-way ANOVA with Tukey's post hoc test. ns indicates no statistical difference.

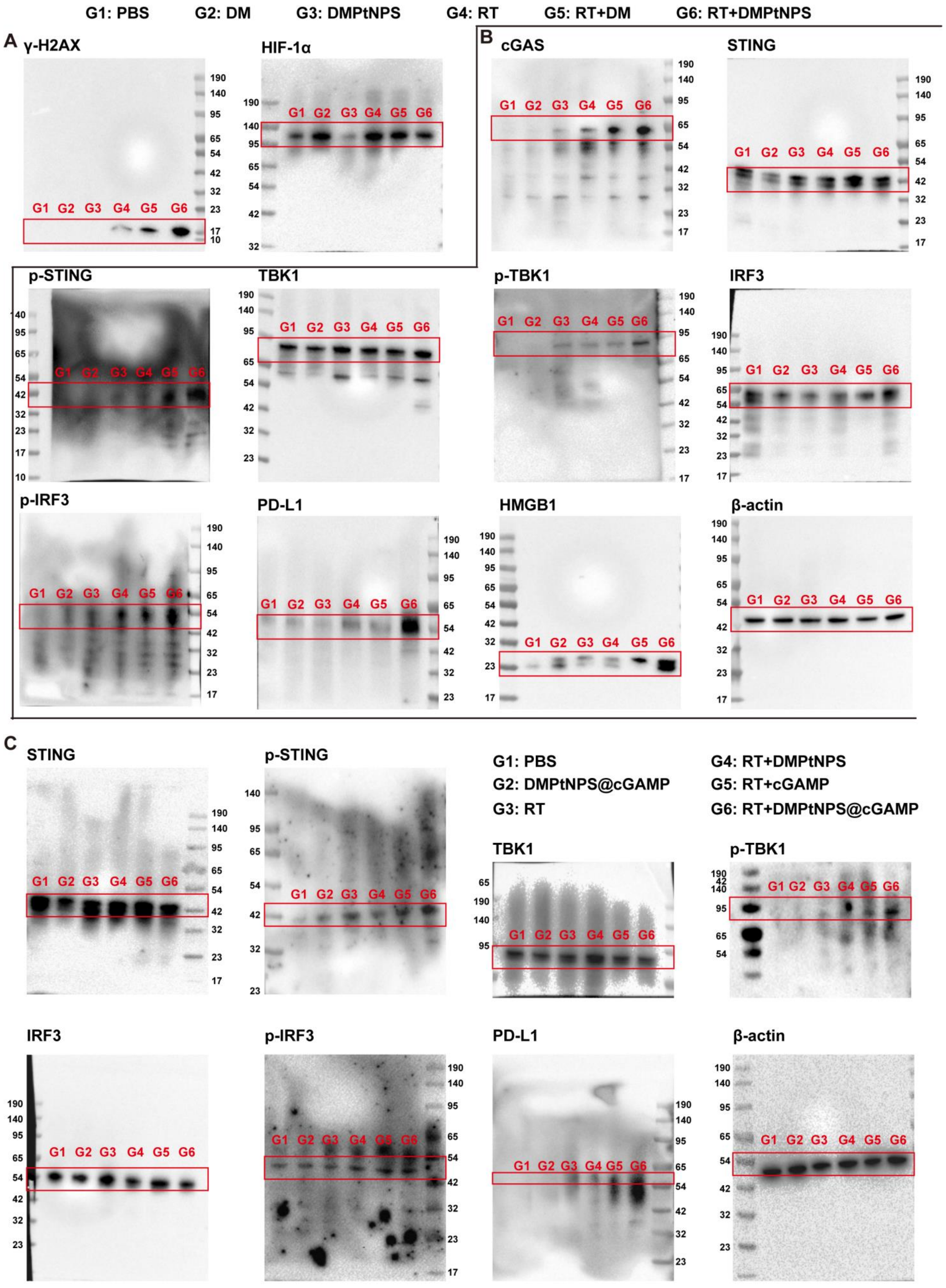
G1: PBS
G2: DM
G3: DMPtNPS
G4: RT
G5: RT+DM
G6: RT+DMPtNPS
A
γ-H2AX
HIF-1α
B
cGAS
STING
p-STING
TBK1
p-TBK1
IRF3
p-IRF3
PD-L1
HMGB1
β-actin
G1 G2 G3 G4 G5 G6
C
STING
p-STING
G1: PBS
G2: DMPtNPS@cGAMP
G3: RT
G4: RT+DMPtNPS
G5: RT+cGAMP
G6: RT+DMPtNPS@cGAMP
TBK1
p-TBK1
IRF3
p-IRF3
PD-L1
β-actin

Figure S15. Images of original uncropped western blots of Fig. 4G (A), Fig. 5A (B) and of Fig. 7B (C). The red rectangles outline the images used in the listed figures.

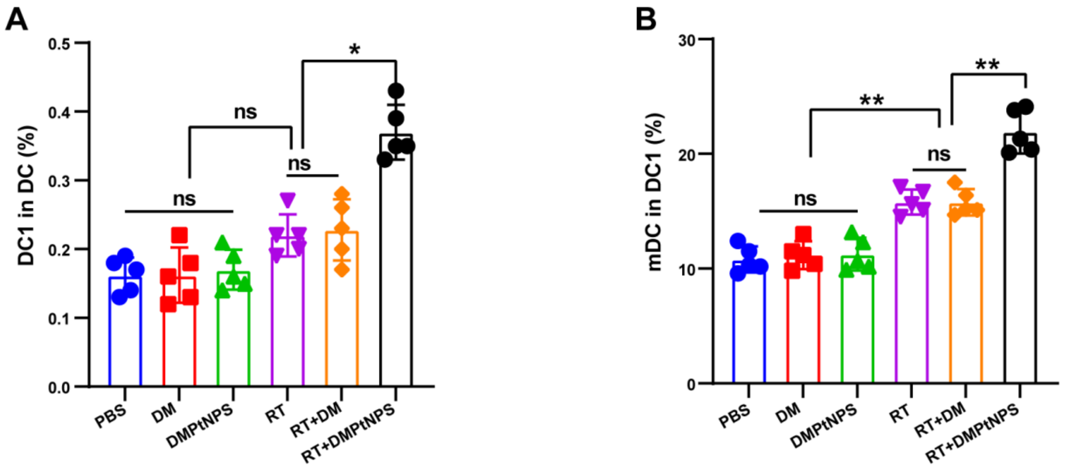

Figure S16. The quantification of DC1 (A) and mature DC cells (B) in tumors after indicated treatments. Data are presented as mean ± SD, n=5. Statistical significance was calculated by one-way ANOVA with Tukey's post hoc test. ns indicates no statistical difference, *$P < 0.05$, **$P < 0.01$.

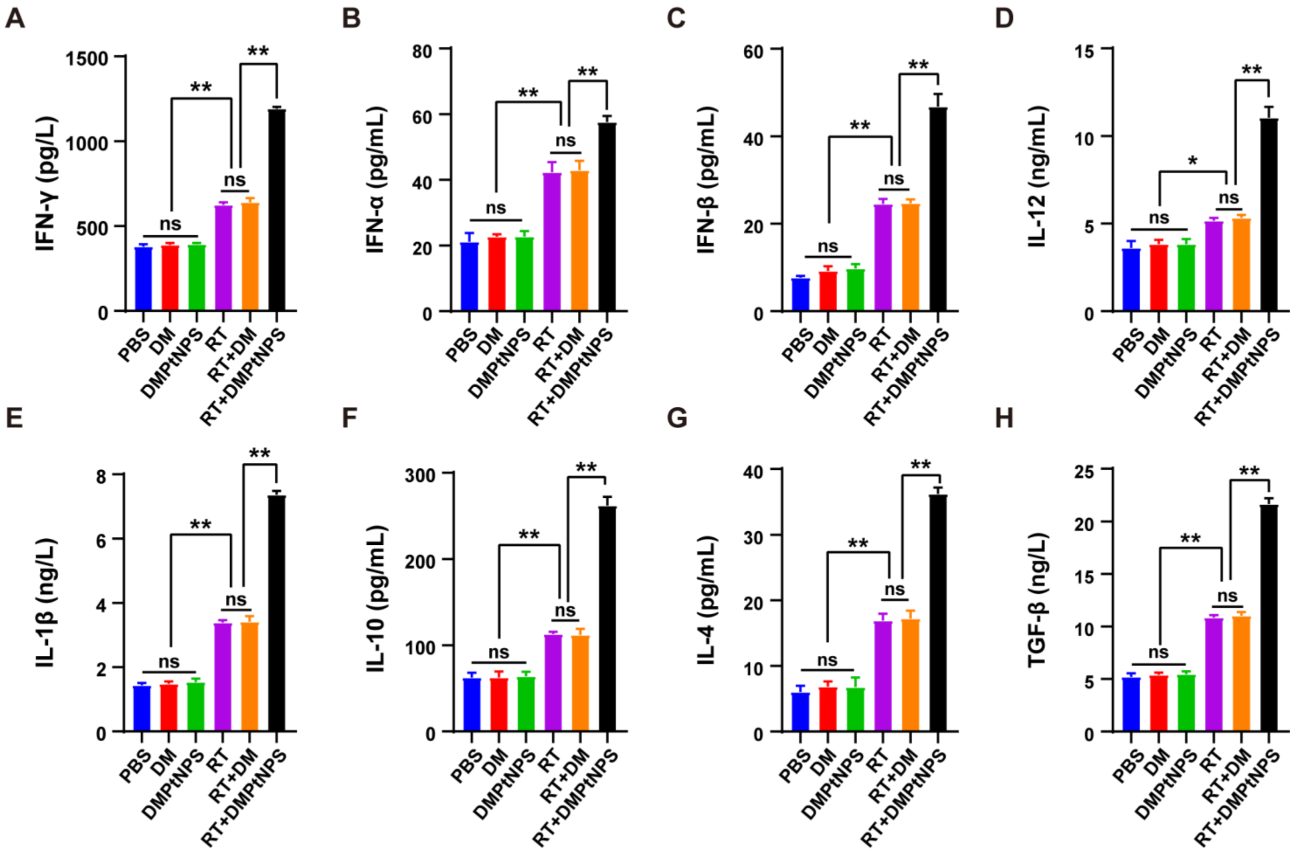

Figure S17. Cytokines levels of IFN-γ (A), IFN-α (B), IFN-β (C), IL-12 (D) IL-1β (E), IL-10 (F), IL-4 (G), TGF-β (H) in tumors after indicated treatments. Data are presented as mean ± SD, n=5. Statistical significance was calculated by one-way ANOVA with Tukey's post hoc test. ns indicates no statistical difference, $^{*}P < 0.05$, $^{**}P < 0.01$.

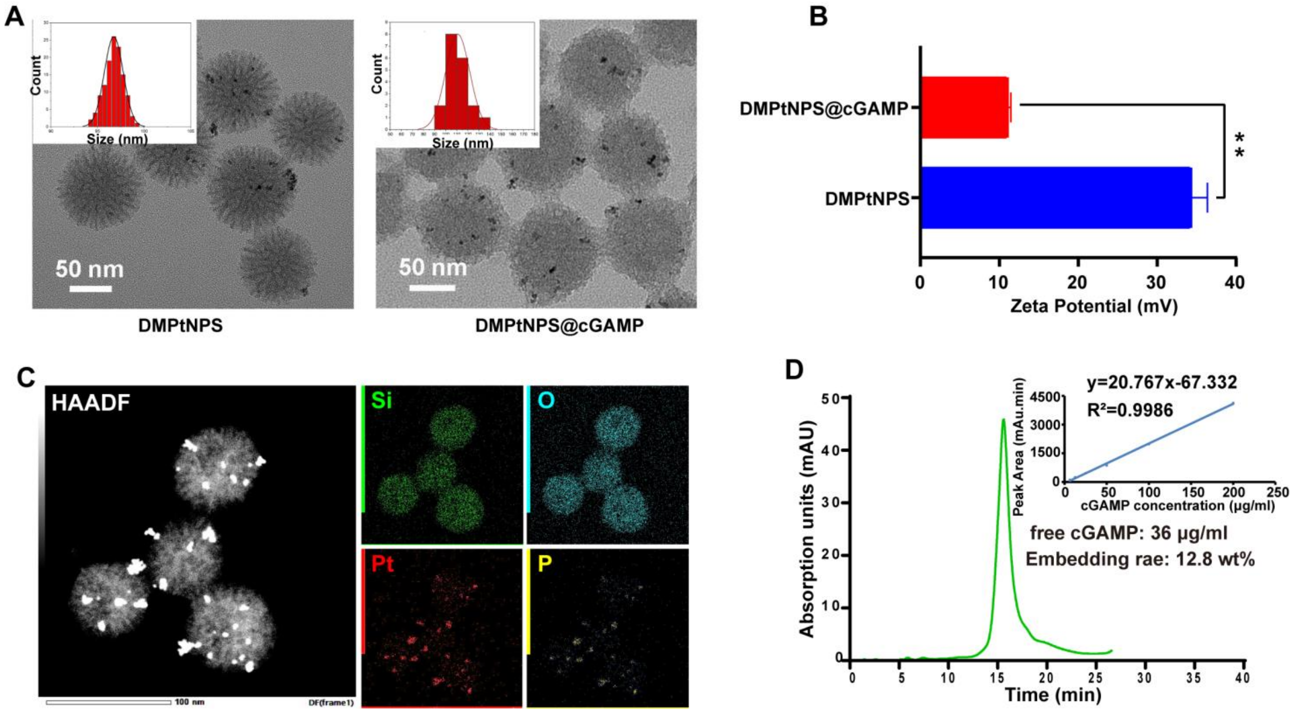

Figure S18. (A) TEM images of DMPtNPS and DMPtNPS@cGAMP nanoparticles. The inset pictures show the size distribution of DMPtNPS and DMPtNPS@cGAMP as calculated by ImageJ software. (B) Zeta potential of DMPtNPS and DMPtNPS@cGAMP (n = 3). (C) HAADF element mapping of DMPtNPS@cGAMP. (D) The amount of cGAMP loaded in DMPtNPS@cGAMP was analyzed by high-performance liquid chromatography (HPLC). Data are presented as mean ± SD, n=3. Statistical significance was calculated by one-way ANOVA with Tukey's post hoc test. $**P < 0.01$.

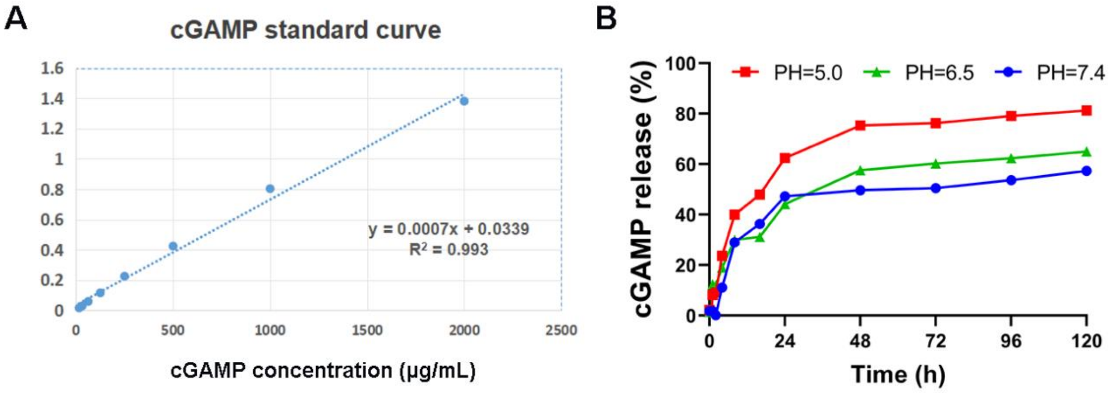

Figure S19. The linear fit of cGAMP and its release from DMPtNPS@cGAMP at different pH conditions as indicated.

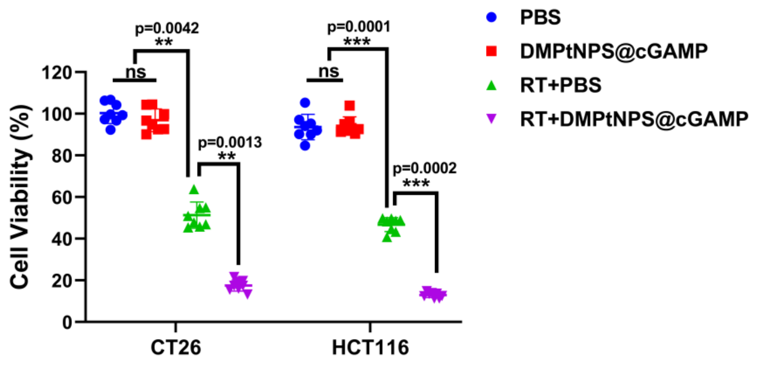

Figure S20. The cytotoxicity of DMPtNPS@cGAMP on CT26 cells and HCT116 cells after indicated treatments *in vitro*. Data are presented as mean ± SD, n=6. Statistical significance was calculated by one-way ANOVA with Tukey's post hoc test. ns indicates no statistical difference, $^{**}P < 0.01$, $^{***}P < 0.001$.

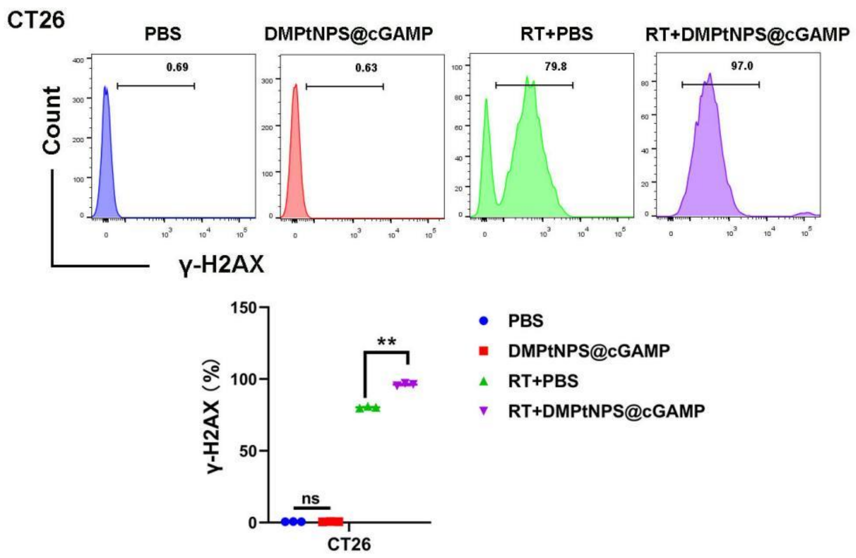

Figure S21. FCM analysis and the γ-H2AX levels in CT26 cells after indicated treatments *in vitro*. Data are presented as mean ± SD, n=3. Statistical significance was calculated by one-way ANOVA with Tukey's post hoc test. ns indicates no statistical difference, $^{**}P < 0.01$.

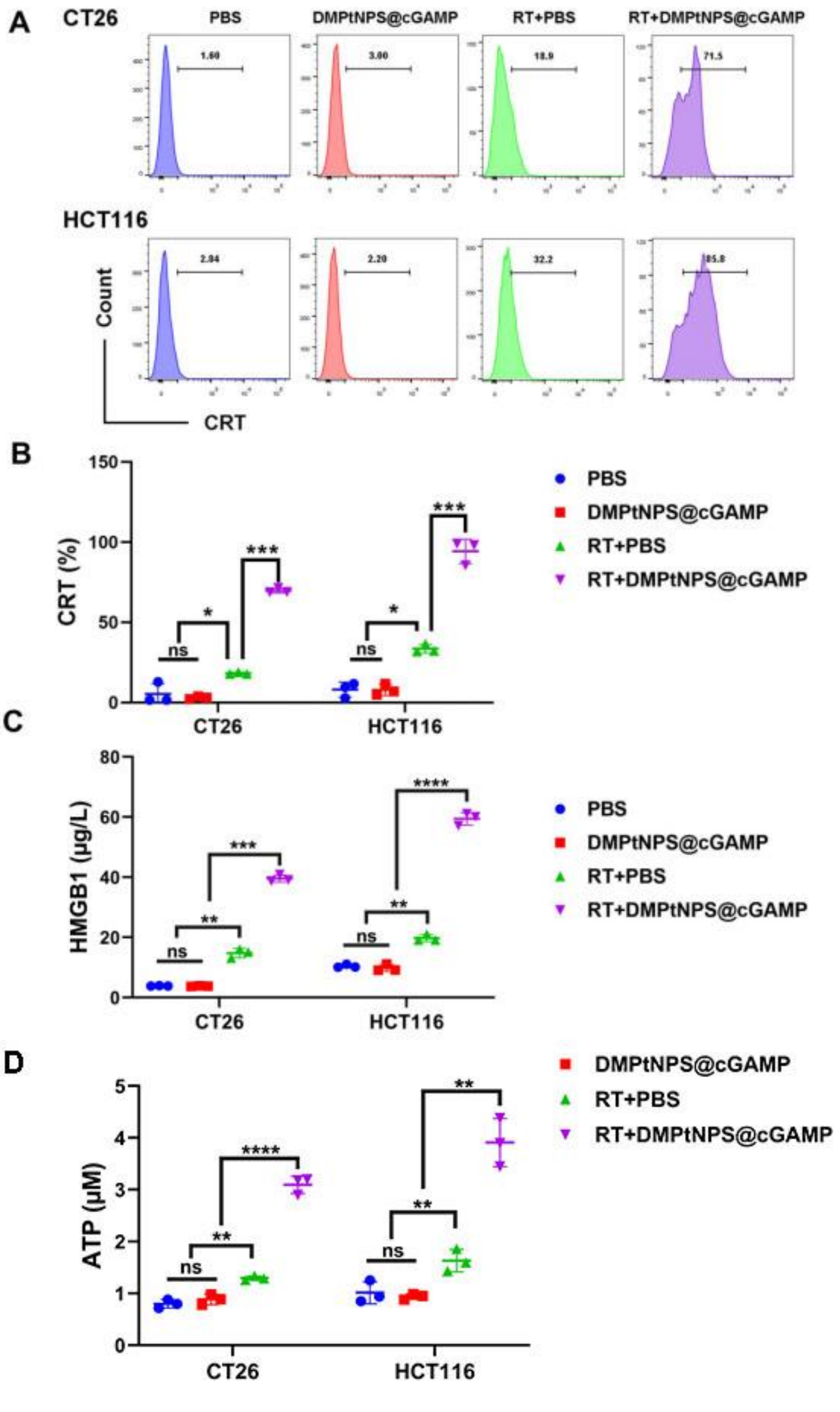

Figure S22. DMPtNPS@cGAMP enhanced RT-induced immunogenic cell death in vitro. (A) FCM analysis of CRT exposure in CT26 cells and HCT116 cells after indicated treatments. (B) Quantitation of CRT in CT26 cells and HCT116 cells after indicated treatments. (C) HMGB1 levels in CT26 cells and HCT116 cells after indicated treatments. (D) Quantitative analysis of

extracellular ATP in CT26 cells and HCT116 cells after indicated treatments. Data are presented as mean ± SD, n=3. Statistical significance was calculated by one-way ANOVA with Tukey's post hoc test. ns indicates no statistical difference, $*P < 0.05$, $**P < 0.01$, $***P < 0.001$, $****P < 0.0001$.

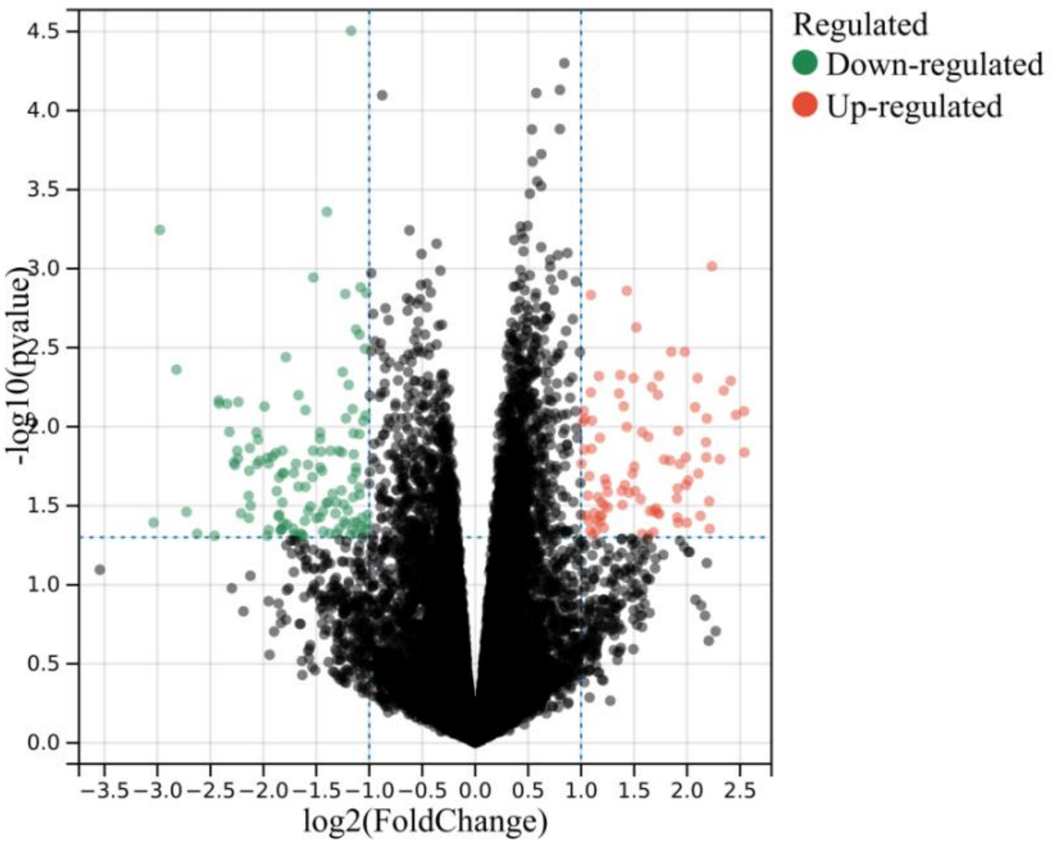

Figure S23. The differentially expressed genes visualized by volcano plot between DMPtNPS + RT group and DMPtNPS@cGAMP+RT, (n = 4).

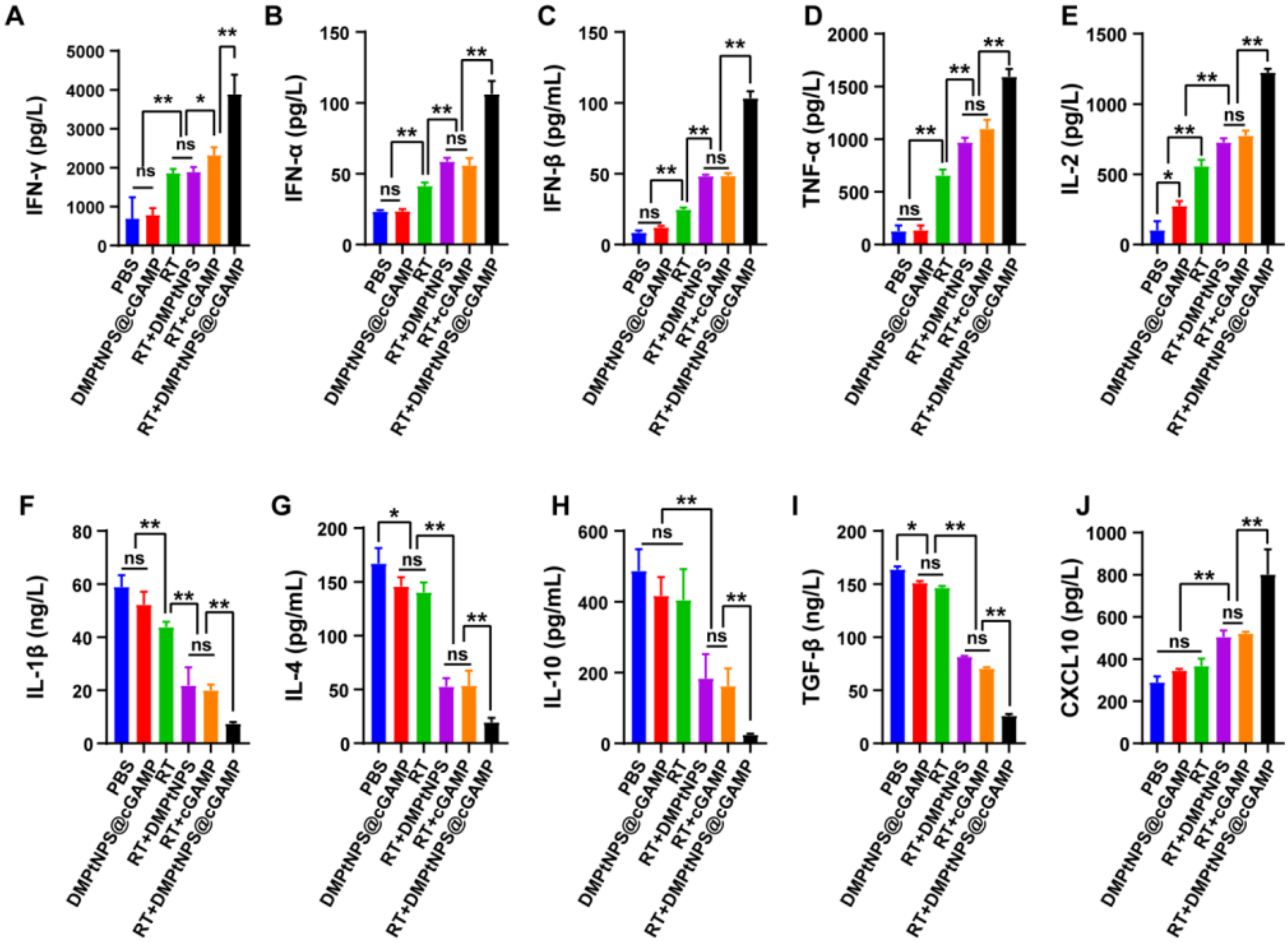

Figure S24. Cytokines levels of IFN-γ (A), IFN-α (B), IFN-β (C), TNF-α (D), and IL-2 (E), pro-tumor cytokines IL-1β (F), IL-4 (G), IL-10 (H), TGF-β (I) and CXCL10 (J) in tumors after indicated treatments. Data are presented as mean ± SD, n=5. Statistical significance was calculated by one-way ANOVA with Tukey's post hoc test. ns indicates no statistical difference, $*P < 0.05$, $**P < 0.01$.

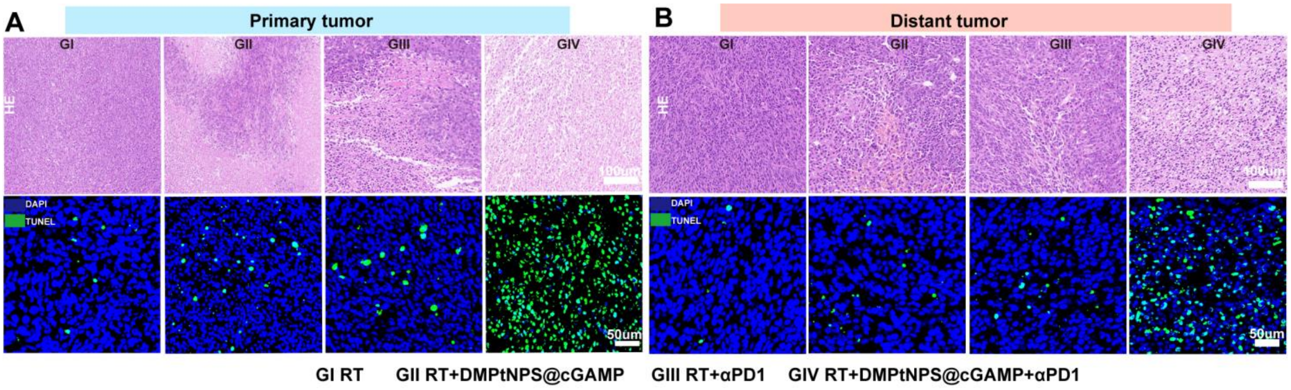

Figure S25. H&E staining (Scale bar, 100 μm) and Tunnel staining (green, Scale bar, 50 μm) of primary tumors (A) and distant tumors (B) after indicated treatments at day 5th.

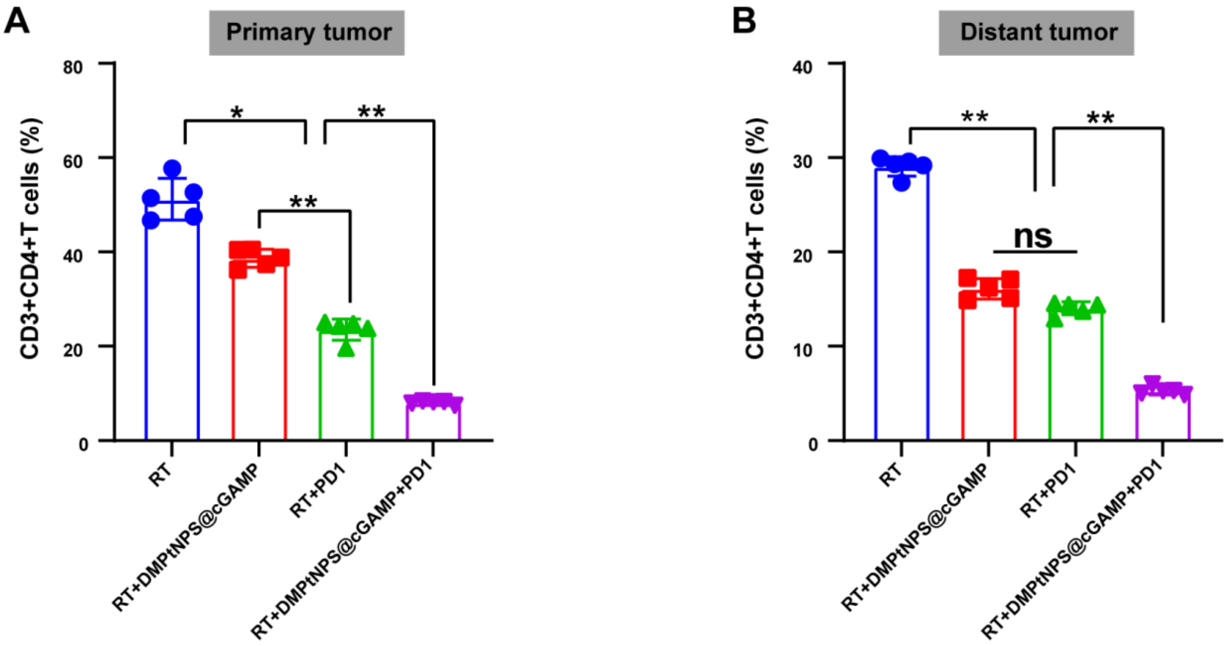

Figure S26. FCM analysis of infiltrated CD4+T cells in the primary tumors (A) and distant tumors (B) after indicated treatment. Data are presented as mean ± SD, n=5. Statistical significance was calculated by one-way ANOVA with Tukey's post hoc test. ns indicates no statistical difference, $^{*}P < 0.05$, $^{**}P < 0.01$.

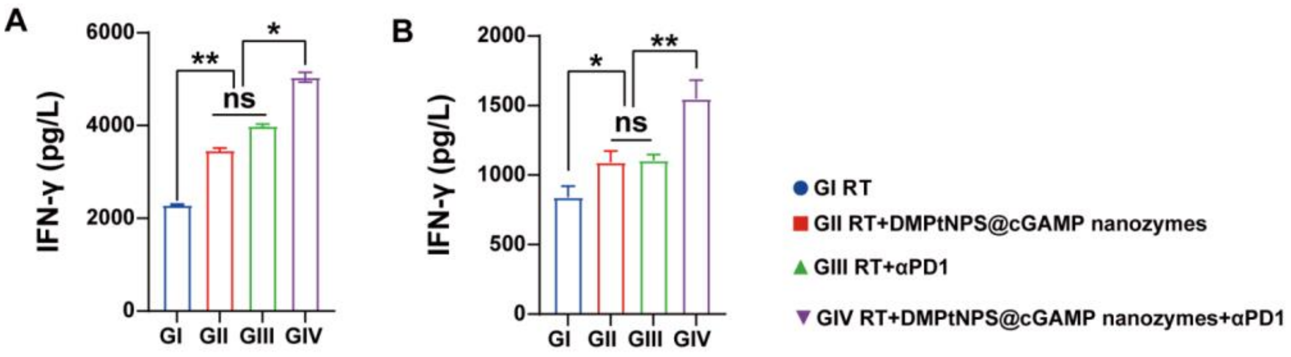

Figure S27. The levels of IFN-γ in primary tumors (A) and distant tumors (A) after indicated treatment. Data are presented as mean ± SD, n=5. Statistical significance was calculated by one-way ANOVA with Tukey's post hoc test. ns indicates no statistical difference, $*P < 0.05$, $**P < 0.01$.

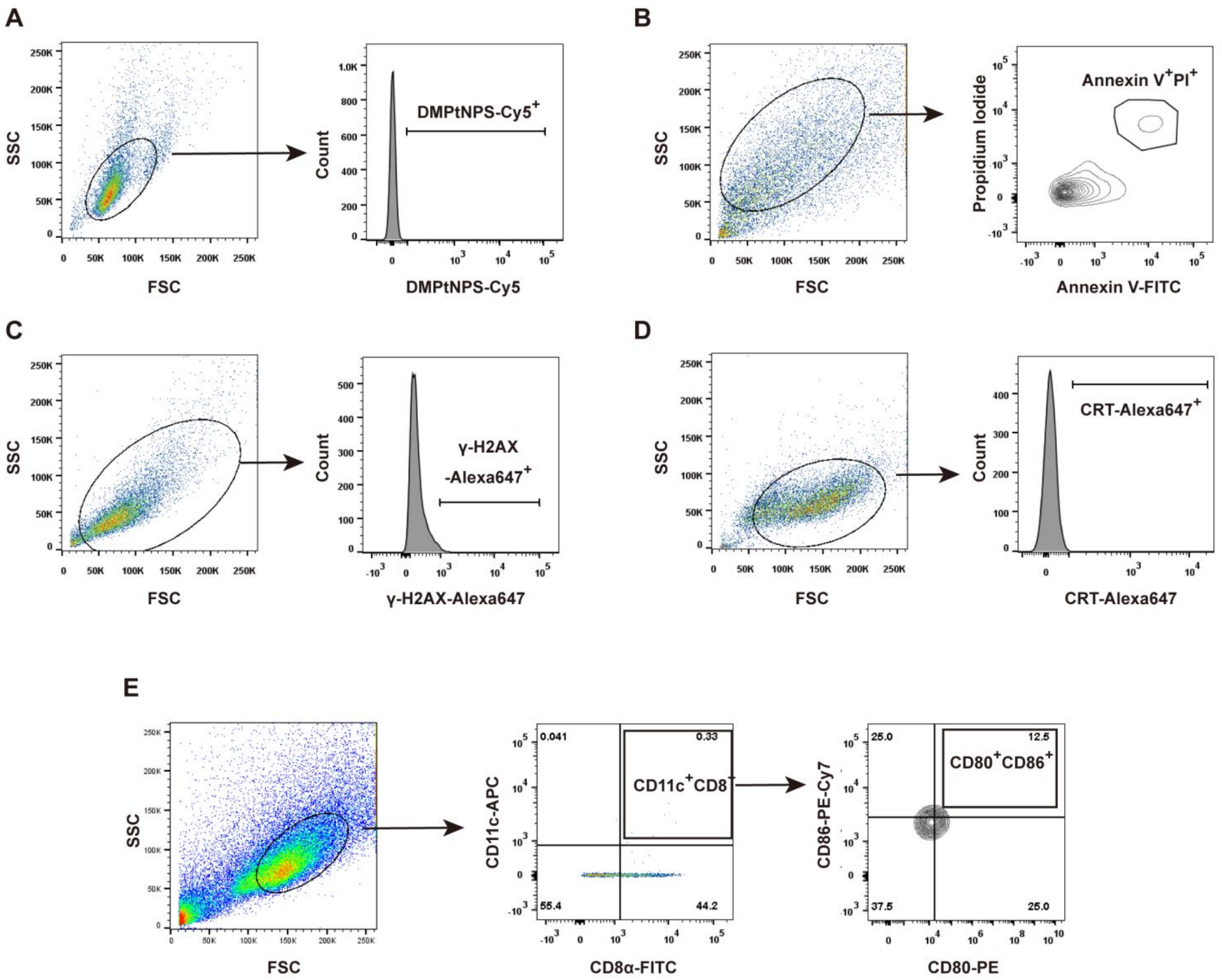

Figure S28. Gating strategies used for *in vitro* studies. Gating strategy for the DMPtNPS uptake in Supplement Fig. 4 (A), the apoptosis staining in Fig. 3I (B), γ-H2AX staining in Fig. 3J (C), CRT staining in Supplement Fig. 7A and B (D), and DC staining in Fig. 4 F (E).

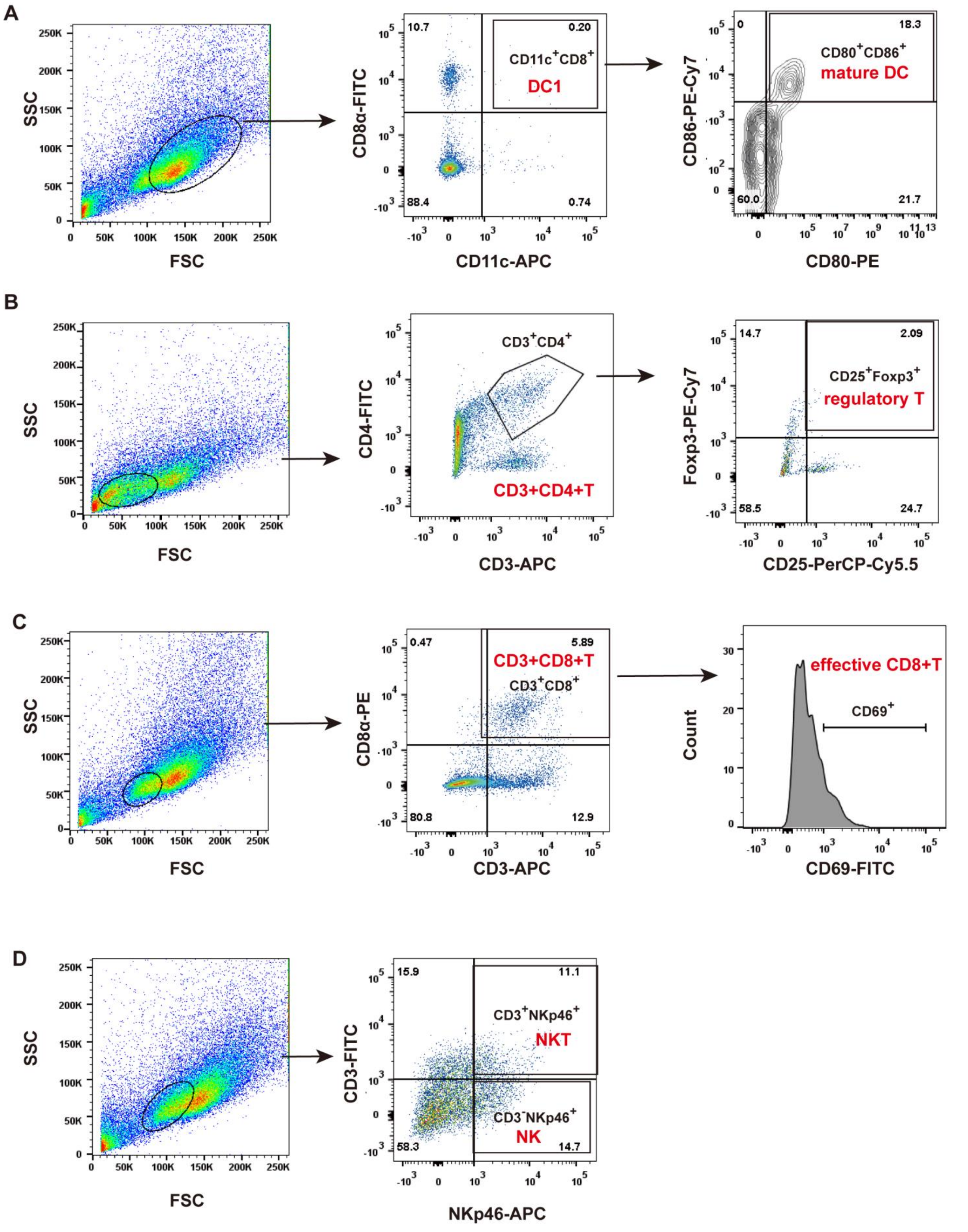

Figure S29. Gating strategies used for *in vivo* studies. Gating strategy for DC1 and mature DC (A), $CD3^+CD4^+$T subgroup and regulatory T subgroup (B), $CD3^+CD8^+$T subgroup and $CD69^+CD8^+$T subgroup (C), and $CD3^-NKp46^+$cells and $CD3^+NKp46^+$cells (D).
